# Influence of Housing Temperature and Genetic Diversity on Allogeneic T Cell-Induced Tissue Damage in Mice

**DOI:** 10.3390/pathophysiology30040039

**Published:** 2023-11-20

**Authors:** Josue Enriquez, Brianyell McDaniel Mims, Stephanie Stroever, Andrea Pires dos Santos, Yava Jones-Hall, Kathryn L. Furr, Matthew B. Grisham

**Affiliations:** 1 Department of Microbiology and Immunology, University of Gothenburg, 40530 Gothenburg, Sweden; 2 Department of Oral Health Sciences, Medical University of South Carolina, Charleston, SC 29425, USA; 3 Department of Medical Education, Texas Tech University Health Sciences Center, Lubbock, TX 79430, USA; 4 Department of Comparative Pathobiology, College of Veterinary Medicine, Purdue University, West Lafayette, IN 47907, USA; 5 Department of Veterinary Pathobiology, School of Veterinary Medicine and Biomedical Sciences, Texas A&M University, College Station, TX 77843, USA; 6 Department of Immunology and Molecular Microbiology, Texas Tech University Health Sciences Center, Lubbock, TX 79430, USA

**Keywords:** bone marrow failure, acute graft vs. host disease, spleen hypoplasia, T cells, reduced intensity conditioning, anemia, cytopenia, outbred mice, housing temperature

## Abstract

The objective of this study was to determine how housing temperature and genetic diversity affect the onset and severity of allogeneic T cell-induced tissue damage in mice subjected to reduced intensity conditioning (RIC). We found that adoptive transfer of allogeneic CD4^+^ T cells from inbred donors into sub-lethally irradiated inbred recipients (I→I) housed at standard housing temperatures (ST; 22–24 °C) induced extensive BM and spleen damage in the absence of injury to any other tissue. Although engraftment of T cells in RIC-treated mice housed at their thermo-neutral temperature (TNT; 30–32 °C) also developed similar BM and spleen damage, their survival was markedly and significantly increased when compared to their ST counterparts. In contrast, the adoptive transfer of allogeneic T cells into RIC-treated outbred CD1 recipients failed to induce disease in any tissue at ST or TNT. The lack of tissue damage was not due to defects in donor T cell trafficking to BM or spleen but was associated with the presence of large numbers of B cells and myeloid cells within these tissues that are known to contain immunosuppressive regulatory B cells and myeloid-derived suppressor cells. These data demonstrate, for the first time, that housing temperature affects the survival of RIC-treated I→I mice and that RIC-conditioned outbred mice are resistant to allogeneic T cell-induced BM and spleen damage.

## 1. Introduction

Transplant of allogeneic hematopoietic stem cells is a potentially curative treatment for malignant and nonmalignant hematological diseases [1,2,3,4]. For this procedure, hematopoietic stem cells (HSCs) from peripheral or umbilical cord blood or bone marrow (BM) from healthy donors are transplanted into patients, where these stem cells differentiate and expand into new and healthy blood and immune cells. In general, healthy/younger patients receive standard myeloablative conditioning prior to hematopoietic stem cell transplantation (HSCT) in order to create immunological space to facilitate engraftment, proliferation and differentiation of donor HSCs into the cellular elements of blood. In an attempt to increase the number of potential donors, both related and unrelated donors expressing one copy of chromosome 6 containing the HLA loci are used for HSCT. The use of these haploidentical (i.e., *allogeneic*) donors has allowed >95% of all patients who require HSCT to receive this treatment [5]. Unfortunately, 40–50% of patients receiving allogeneic HSCT will develop a potentially life-threatening disease called acute graft vs. host disease (aGVHD) [3,6]. This donor T cell-mediated disease typically targets the intestines, liver and/or skin [3,6]. Preclinical and clinical studies demonstrate that bone marrow (BM) and spleen are particularly sensitive to aGVHD-induced injury as relatively small numbers of allogeneic CD4^+^ T cells are capable of damaging these hematopoietic tissues [7,8,9,10,11,12]. Damage to these tissues creates defective T and B cell-mediated immunity as well as thrombocytopenia and anemia [10,12,13,14]. This immuno-deficient state remarkedly increases the risk of infections and bleeding and is associated with considerable morbidity and mortality that may account for ~30% of patient deaths with aGVHD [10,12,13]. Much of what is known regarding the immunopathogenesis of aGVHD has come from studies using mouse models of disease [3,6,15,16,17]. These preclinical studies have been instrumental in helping to define many of the immunopathological mechanisms responsible for disease development and revealing novel therapeutic targets for more effective drug development. Despite these promising findings, the translation of these experimental data into more effective therapeutics for the treatment of patients has been poor [17,18,19,20].

The reasons for the poor bench-to-bedside translation are not completely clear; however, recent studies have suggested that both extrinsic and intrinsic factors inherent in mouse models of aGVHD may alter disease pathogenesis when compared to human aGVHD, thereby affecting the translatability of these models [20].

For example, the large majority of mouse models of aGVHD use lethal, total body irradiation (TBI) to produce myeloablation prior to BM and T cell transfer [16,17]. Lethal TBI induces indiscriminate damage to several different tissues and organ systems that respond with the upregulation of numerous inflammatory cytokines, chemokines and chemokine receptors that promote the recruitment and activation of alloreactive T cells to the gut, liver, skin, lung, thymus, BM, and/or spleen [8,10,13,15,16,17,21,22,23,24,25,26,27]. This protocol contrasts with more current, pretransplant conditioning protocols for patients who receive nonmyeloablative, reduced intensity conditioning to decrease tissue injury and the consequent development of aGVHD [2,17,28]. Another important extrinsic factor that is receiving increased attention in preclinical studies is animal care housing temperature. All animal care facilities in the U.S. house mice at a standard temperature (ST) of 22–24 °C, which is below murine thermoneutral temperature (TNT) of 30–32 °C [29,30,31,32]. Vivaria ST housing is known to create mild cold stress that may alter murine immune responses to different allo- or autoantigens when compared with mice housed at TNT [29,30,31,32].

In addition to extrinsic factors, a lack of genetic diversity may represent an important intrinsic factor that may limit bench-to-beside translation in mouse models of aGVHD [20]. Virtually all mouse models of aGVHD use *inbred* strains of mice as surrogates for genetically diverse humans. There is no question that the use of inbred mice has been critical to our fundamental understanding of the role of the immune system in health and disease. However, it is not clear whether immune responses of inbred mice recapitulate the diverse immune responses found in humans. Several studies have reported that specific immune responses and disease phenotypes may be quite different in inbred vs. genetically diverse *outbred* mice [33,34,35,36,37,38]. To our knowledge, no studies have been reported using outbred mice to study the immuno-pathogenesis of aGVHD. When taken together, it is possible that one or more of the extrinsic and/or intrinsic factors described above may affect outcomes in preclinical studies that limit their translation to patient care. The objective of this study was to determine how housing temperature and genetic diversity affect the development of aGVHD in mice subjected to reduced intensity conditioning (RIC). We found that engraftment of allogeneic CD4^+^ T cells from inbred donors into inbred recipients subjected to RIC and housed at ST or TNT induced dramatic BM failure and spleen hypoplasia that were associated with severe anemia and cytopenia. Surprisingly, the survival of mice housed at their TNT was significantly increased when compared to mice housed at ST. In contrast, engraftment of RIC-treated outbred CD1 mice with allogeneic inbred CD4^+^ T cells failed to induce disease when housed at ST or TNT, suggesting that outbred mice are resistant to developing aGVHD. The potential mechanisms involved in the selective, T cell-mediated damage to BM and spleen in inbred mice and resistance to disease induction in outbred mice are discussed.

## 2. Materials and Methods

### 2.1. Mice

Eight- to ten-week old *inbred* male C57Bl/6J (Bl6; H2^b^) mice, C57Bl/6.SJL*Ptprc^a^ Pepc^b^*/J (Bl6/CD45.1; H2^b^) mice and C57Bl6(C)-*H2*-*Ab1^bm12^*/KhEgJ (BM12; H2^bm12^) mice were purchased from Jackson Laboratory (Bar Harbor, ME, USA), whereas male, age-matched *outbred* CD1 (H2^b,k,p,q^) mice were purchased from Charles River, Inc (Wilmington, MA, USA). All mice were housed in filter top cages in the LARC facility at 22–24 °C under specific pathogen-free (SPF) barrier conditions. All experimental procedures involving the use of animals were reviewed and approved by the Institutional Animal Care and Use Committee of TTUHSC and performed according to the criteria outlined by the National Institutes of Health. For some experiments, mice were housed at their thermo-neutral temperature (TNT) of 30–32 °C in an Allentown NexGen thermal recovery incubator. This incubator is capable of holding 12 cages, allowing for the housing of mice at different temperatures (including 30–32 °C) in a well-controlled environment.

### 2.2. Induction of aGVHD Using Inbred and Outbred Mice

The objective of the first series of studies was to establish and characterize two mouse models of aGVHD-induced bone marrow (BM) and spleen damage using allogeneic inbred donors and inbred recipients subjected to reduced intensity conditioning (RIC; *sublethal irradiation*). Using a modification of the method published by Chen et al. [39], flow-purified CD4^+^CD25^−^ T conventional (Tconv) cells were isolated from the spleens of Bl6 or BM12 donor mice and then injected (*i.p.*) into *sub-lethally irradiated* (450 cGy) BM12 or Bl6 mice, respectively (Bl6→BM12, BM12→Bl6). The MHC class II (MHC II) proteins of BM12 mice differ from those of Bl6 animals by only 3 amino acids, which are sufficient to drive aGVHD [40]. Tconv cells were flow-purified using fluorescence-activated cell sorting (FACS) as described in our previously published protocols [9,41]. Approximately 80–85% of the flow-purified Tconv cells expressed the naïve phenotypic markers of CD4^+^CD62L^+^CD44^low^CD25^−^. Engraftment of Bl6 or BM12 T cells into sub-lethally irradiated Bl6 or BM12 recipients, respectively, served as the syngeneic control groups for these studies. Disease was evaluated in both groups of mice housed at standard temperature (ST; 22–24 °C). We next wished to evaluate whether housing temperature affected the onset and/or severity of BM failure and spleen hypoplasia. To do this, we injected (*i.v.*) varying numbers of Bl6/CD45.1 Tconv cells into sub-lethally irradiated BM12 recipients (Bl6/45.1→BM12) that were housed at ST or thermoneutral temperature (TNT; 30–32°C). For the TNT studies, BM12 mice were acclimated for 7 days at TNT prior to sub-lethal irradiation and adoptive transfer of T cells. Mice were then subjected to sub-lethal irradiation followed by adoptive transfer of varying numbers of Bl6/CD45.1 Tconv cells and returned to their cages, where they were maintained at their TNT for an additional 3–4 weeks. Our last series of studies was designed to determine whether adoptive transfer of allogeneic T cells from inbred donors into sub-lethally irradiated *outbred* recipients induced immune-mediated BM failure and/or spleen hypoplasia. Varying numbers of flow-purified Tconv cells isolated from the spleens of Bl6/45.1 or BM12 donor mice were injected (*i.v.*) into sub-lethally irradiated (500 cGy) CD1 recipients. For all experiments described above, mice were weighed 2–3 times weekly and evaluated for signs of disease. Mice presenting with severe disease as evidenced by lethargy, kyphosis (hunched appearance) and/or weight loss ≥20% of their original weight were designated as moribund and euthanized.

### 2.3. Complete Blood Cell Analyses and Plasma Cytokine Quantification

Prior to euthanasia, aliquots of blood were collected from each mouse to perform the following analyses: (a) Quantification of circulating numbers of total leukocytes, lymphocytes, granulocytes and platelets as well as hemoglobin concentration and hematocrit were determined using the complete blood cell counts (CBC) software (version 2.7.6) associated with the Heska HemaTrue Veterinary Hematology Analyzer; and (b) Quantification of plasma cytokine concentrations using the Legendplex^TM^ multiplex bead assay that quantifies 13 different cytokines including TNF-α, IL-1α, IL-1β, IFN-γ, IL-6, IL-17A, IL-23, MCP-1, IL-12p70, IL-10, IL-27, IFN-β, and GM-CSF (BioLegend, Inc., San Diego, CA, USA).

### 2.4. Immune Cell Analysis

Following euthanasia, the numbers and phenotypes of the T, B, myeloid, and NK cells in the spleen and bone marrow were quantified using flow cytometry as we previously described [9]. Briefly, single-cell suspensions from spleens and femoral BM were suspended in Tris-buffer with ammonium chloride (ACT) to lyse red blood cells and then resuspended in FACs buffer (PBS containing 4% fetal calf serum). A hemocytometer was used to quantify the total number of live (i.e., trypan blue-excluding) BM and spleen cells. Cells were stained with CD4 PE-Cy7 (GK1.5), CD8 V450 (53–6.7), CD25 PE (PC61), CD19 PerCP-Cy5.5 (1D3), CD335 Alexa Fluor647 (NK p46)(29A1.4) and CD11b FITC (m170) and analyzed by flow cytometry [42].

### 2.5. Tissue Preparation for Blinded Histological Evaluation

Following euthanasia, femurs, spleen, colon, skin, liver and lung were removed from each animal, fixed in phosphate-buffered formalin and stored at 4 °C. Tissues were embedded in paraffin, sectioned (5 μm) and stained with hematoxylin and eosin. Blinded histological scoring of each tissue section was performed using our established scoring criteria. A detailed description of the scoring criteria used for each tissue can be found in reference [43].

### 2.6. Statistical Analyses

Statistical analyses for the following three determinations were performed using GraphPad Prism software (version 6): (a) Student’s T test for significant differences between 2 groups, (b) one-way analysis of variance and Tukey’s multiple comparison post hoc test for significant differences among 3 or more groups and (c) Kaplan–Meir survival plots for significant differences between 2 or more groups. Statistical analysis of the time-dependent changes in animal body weight was performed using Stata/MP version 17.0.

## 3. Results

### 3.1. Induction and Characterization of Allogeneic T Cell-Mediated Tissue Damage in Inbred Mice

**Figure 1:**
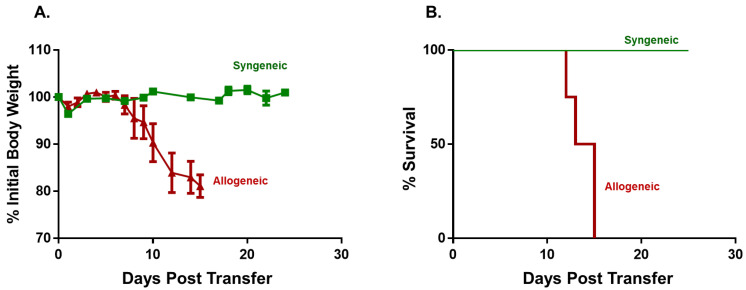
Adoptive transfer of allogeneic CD4^+^ T cells from BM12 donors into Bl6 recipients induces lethal graft vs. host disease (GVHD). Flow sorted, allogeneic CD4^+^CD25^−^ T cells from BM12 donors (20,000 T cells/gram body weight; 20k T cells/gbw) were injected (*i.p.*) into sub-lethally irradiated Bl6 recipients (BM12→Bl6). For the syngeneic group, sub-lethally irradiated Bl6 mice were injected (*i.p.*) with CD4^+^CD25^−^ T cells from Bl6 donors (20k/gbw). (**A**) Body weights of mice in the two groups at different times following injection of T cells. Data represent the mean ± SEM. *p* < 0.029 at day 11 for syngeneic vs. allogeneic group. (**B**) Kaplan–Meir survival plots of both groups. Mice exhibiting severe disease as evidenced by lethargy, kyphosis (hunched appearance) and/or weight loss ≥20% of their original weight were designated as moribund and euthanized. *p* < 0.01 for syngeneic vs. allogeneic group. The starting number of mice in the syngeneic and allogeneic groups was N = 4.

The objective of the first series of studies was to establish and characterize two mouse models of aGVHD that exhibit selective damage to the BM and spleen following adoptive transfer of allogeneic inbred donor T cells into inbred recipients subjected to reduced intensity conditioning (RIC, i.e., *sublethal irradiation*). We found that injection (*i.p.*) of flow-sorted allogeneic BM12 CD4^+^CD25^−^ T conventional (Tconv) cells (20,000 T cells/gram body weight i.e., 20k T cells/gbw) into sub-lethally irradiated (450 cGy) Bl6 recipients (BM12→Bl6) housed at standard animal care temperature (ST; 22–24 °C) induced severe disease such that mice did not survive beyond day 15 post-injection (Figure 1A,B). In contrast, syngeneic mice (Bl6→Bl6) remained healthy and did not develop any signs of aGVHD, with all mice surviving the 25-day observation period (Figure 1A,B).

**Figure 2:**
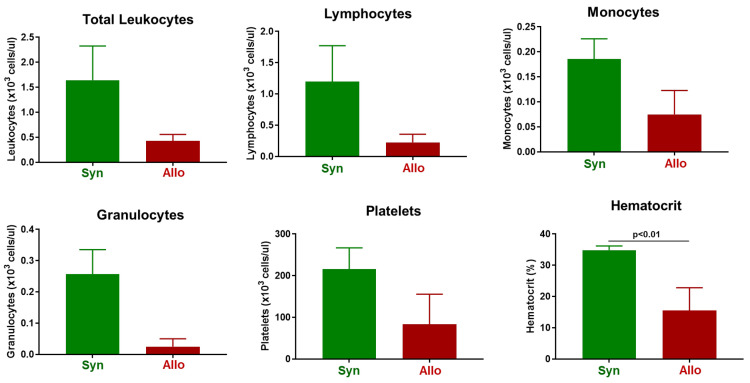
Complete blood cell counts (CBC) in syngeneic and allogeneic mice. CBC analysis was quantified from EDTA-treated whole blood from each mouse using the CBC software associated with the Heska HemaTrue Veterinary Hematology Analyzer at 4 weeks post T cell transfer for syngeneic mice and prior to euthanasia for allogeneic mice. Each syngeneic and allogeneic group has are N = 4 mice. Data represent the mean ± SEM.

Complete blood cell counts (CBC) revealed that while circulating numbers of total leukocytes, lymphocytes, monocytes, granulocytes and platelets were reduced in allogeneic vs. syngeneic mice, these differences were not statistically significant (Figure 2). However, we did observe severe anemia as assessed by a large and significant reduction in hematocrit in mice engrafted with allogeneic T cells (Figure 2).

**Figure 3:**
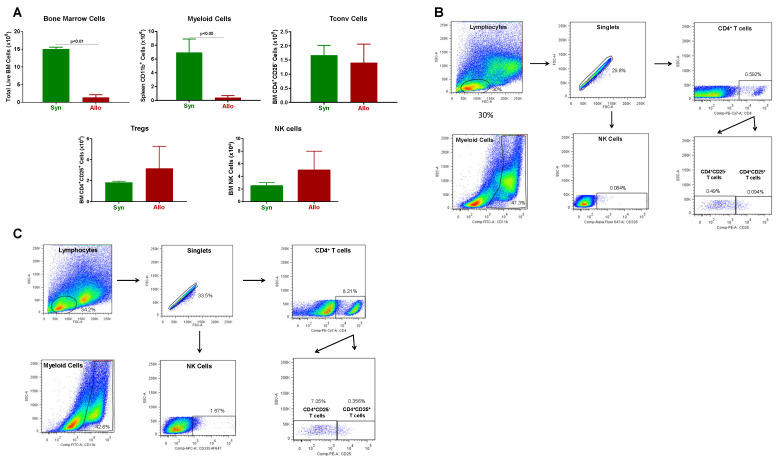
Bone marrow analysis in syngeneic and allogeneic mice. (**A**) Total bone marrow (BM) cell numbers and BM-residing immune cells were quantified at 4 weeks post T cell transfer for syngeneic mice and prior to euthanasia for allogeneic mice described in Figure 1. Myeloid cells (CD11b^+^), Conventional T cells (Tconv cells; CD4^+^CD25^−^), regulatory T cells (Tregs; CD4^+^CD25^+^), and NK (CD335^+^) cells were quantified by flow cytometry as described in Methods Section 2.4. The number of mice in the syngeneic and allogeneic groups are N = 3 and N = 6 each, respectively. Data represent the mean ± SEM. (**B**,**C**) Representative flow cytometry plots of syngeneic and allogeneic mice, respectively.

In addition, we found a dramatic and significant reduction in the number of BM cells in allogeneic vs. syngeneic mice (Figure 3). The loss of BM cells appeared to be due to a large and significant reduction in the number of myeloid cells in allogeneic vs. syngeneic mice, whereas the numbers of Tconv cells, regulatory CD4^+^CD25^+^ T cells (Tregs) and NK cells were not significantly altered (Figure 3).

The loss of cellularity in the BM was confirmed by blinded histopathological analysis demonstrating remarkable reductions in BM cellularity with little or no hematopoietic tissue present in allogeneic vs. syngeneic mice (Figure S1A).

**Figure 4:**
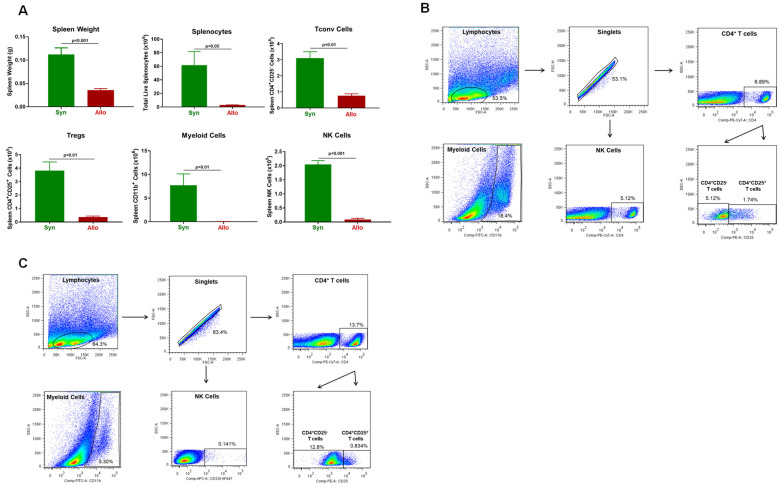
Spleen weight and immune cell analysis in syngeneic and allogeneic mice. (**A**) Spleen weights, splenocyte numbers and spleen-residing immune cells were quantified at 4 weeks post T cell transfer for syngeneic mice and prior to euthanasia for allogeneic mice. Conventional T cells (Tconv cells; CD4^+^CD25^−^), regulatory T cells (Tregs; CD4^+^CD25^+^), Myeloid cells (CD11b^+^), and NK (CD335^+^) cells were quantified by flow cytometry using as described in the Methods Section 2.4. The number of mice in the syngeneic and allogeneic groups are N = 3 and N = 4, respectively. Data represent the mean ± SEM. (**B**,**C**) Representative flow cytometry plots of syngeneic and allogeneic mice, respectively.

Another tissue that is targeted by alloreactive T cells in this model of aGVHD is the spleen [8,14,43,44,45]. We found that spleen weights and splenocyte numbers were remarkably and significantly reduced in allogeneic mice when compared to their syngeneic controls (Figure 4). The loss of splenocytes corresponded to large and significant reductions in the numbers of Tconv cells, Tregs, myeloid cells and NK cells in allogeneic vs. syngeneic controls (Figure 4).

Blinded histopathological analysis of the spleen confirmed pathological changes that were characterized by follicle depletion, loss of white pulp and immune cells and extensive disruption of red pulp (Figure S1B). Of note, engraftment of allogeneic T cells into sub-lethally irradiated recipients failed to induce inflammatory tissue damage in the lungs, liver, colon and skin (body, face, ears) as assessed by blinded histopathological analysis (Figure S2).

In addition, injury to the bone marrow and spleen was associated with large and significant increases in the plasma concentrations of the inflammatory cytokines IFNγ and TNFα, together with marked reductions in plasma levels of GM-CSF (Table 1).

When taken together, these data demonstrate that allogenic CD4^+^ T cells selectively target the BM and spleen where they mediate tissue damage in sub-lethally irradiated recipients. We next wished to determine whether similar BM and spleen injury occurred when donors and recipients were reversed (i.e., Bl6→BM12). Using the same protocol as described above, we found that adoptive transfer (*i.p.*) of allogeneic Bl6 Tconv cells into sub-lethally irradiated BM12 recipients housed at ST induced the time-dependent onset of severe disease such that mice did not survive beyond day 20 post-injection (Figure S3). In contrast, syngeneic BM12→BM12 remained healthy and did not develop any signs of aGVHD during the 25-day observation period (Figure S3). CBC analyses revealed large and significant reductions in the numbers of circulating granulocytes and platelets and a remarkable reduction in hematocrit when compared with their syngeneic controls (Figure S4). In addition, engraftment of allogeneic T cells induced severe BM damage as assessed by the loss of total BM cells, myeloid cells, Tconv, NK cells and B cells (Figures S5 and S6A). Furthermore, Bl6→BM12 mice exhibited remarkable spleen injury as seen by significant reductions in spleen weight, total splenocyte number, Tregs, myeloid cells, B cells and NK cells as well as histological analyses (Figures S6B and S7). Taken together, these data confirm that allogeneic Tconv cells target the BM and spleen in sub-lethally irradiated recipients, where they promote BM and spleen damage.

### 3.2. Onset and Severity of BM and Spleen Damage in Inbred Mice Housed at ST or TNT

**Figure 5:**
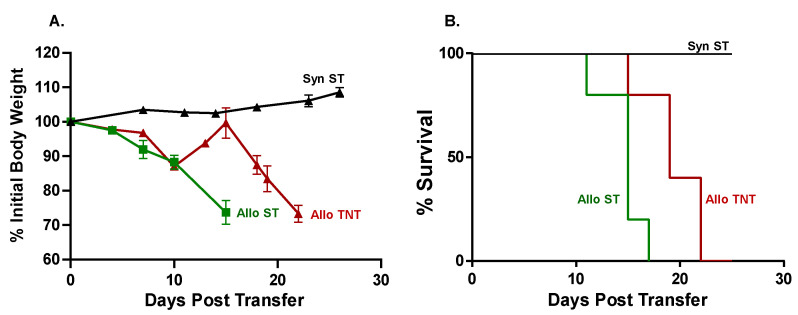
Effects of housing temperature on body weight and survival. Flow-sorted, allogeneic CD4^+^CD25^−^ T cells from CD45.1Bl6 donors (5k T cells/gbw) were injected (*i.v.*) into sub-lethally irradiated BM12 recipients and housed at standard temperature (AlloST) or thermoneutral temperature (AlloTNT). Sub-lethally irradiated BM12 mice injected (*i.v.*) with 5k/gbw CD4^+^CD25^−^ T cells from BM12 donors and housed at ST served as the syngeneic control group (SynST). (**A**) Body weights of mice in the three groups at different times following injection of T cells. (**B**) Kaplan–Meir survival plost of the three groups. Mice exhibiting severe disease as evidenced by lethargy, kyphosis and/or weight loss ≥20% of their original weight were designated as moribund and euthanized. *p* < 0.01 for Syn ST vs. AlloTNT; *p* < 0.01 for SynST vs. AlloST and *p* < 0.05 for AlloST vs. AlloTNT. The starting number of mice in the SynST, AlloST and AlloTNT groups were N = 4, N = 5 and N = 5, respectively.

Having established that *i.p.* administration of allogeneic CD4^+^ T cells selectively targets the BM and spleen in sub-lethally irradiated mice, we next wished to determine whether housing temperature affects the onset and/or severity of disease as well as T cell trafficking to BM and spleen following intravenous administration of T cells. For these studies, we injected (*i.v.*) either 10k or 20k/gbw Bl6/CD45.1 Tconv cells or syngeneic BM12 Tconv cells into sub-lethally irradiated BM12 mice housed at ST. The congenic CD45.1 marker expressed on allogeneic Bl6 T cells was used to identify donor T cells in recipient tissue. We found that *i.v.* administration of 10k or 20k/gbw Bl6/45.1 Tconv cells/gbw into sub-lethally irradiated BM12 recipients housed at ST or TNT greatly accelerated the development of lethal disease such that all mice had to be euthanized within the first 6–9 days post-T cell transfer. In contrast, *i.v.* administration of 10k or 20k/gbw *syngeneic* BM12 T cells did not induce weight loss or reduce survival, with all mice remaining healthy for the 4-week observation period. These preliminary studies prompted us to markedly reduce the numbers of allogeneic T cells in order to more closely approximate the time course for the onset of disease observed in our previous *i.p.* studies. Therefore, sub-lethally irradiated BM12 mice were injected (*i.v.*) with 5k/gbw or 2k/gbw Bl6/45.1 Tconv cells and housed at either ST or TNT. Syngeneic mice housed at ST (SynST) were used as the control group. We found that adoptive transfer of 5k/gbw Bl6/45.1 Tconv cells into sub-lethally irradiated BM12 mice housed at their TNT (AlloTNT) survived for 22 days, whereas mice housed at ST (AlloST) survived for only 17 days, resulting in a significant 29% increase in survival of AlloTNT mice vs. their AlloST counterparts (*p* < 0.05; Figure 5).

**Figure 6:**
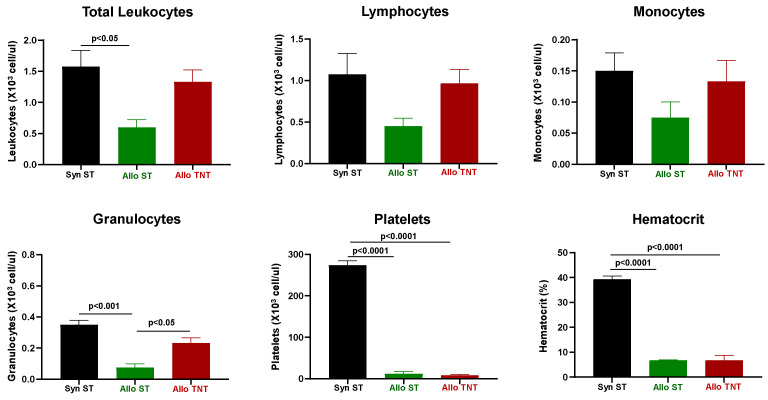
Complete blood cell counts (CBC) in syngeneic and allogeneic mice housed at different temperatures. CBC analysis was quantified from EDTA-treated whole blood from each mouse using the CBC software associated with the Heska HemaTrue Veterinary Hematology Analyzer at 4 weeks post T cell transfer for syngeneic mice and prior to euthanasia for allogeneic mice. The number of mice in each group is presented in Figure 5. Data represent the mean ± SEM.

Engraftment of allogeneic T cells in RIC-treated donors housed at ST significantly reduced the numbers of circulating leukocytes when compared to their syngeneic controls (Figure 6). Although there was a trend for reductions in circulating lymphocytes and monocytes in the AlloST group vs. their SynST controls, these differences were not significant(Figure 6). We did observe large and significant reductions in circulating granulocytes in AlloST mice when compared to the SynST or AlloTNT groups (Figure 6). In addition, marked and significant reductions in platelet numbers and hematocrit were observed in both AlloST and AlloTNT mice when compared to their SynST controls (Figure 6).

**Figure 7:**
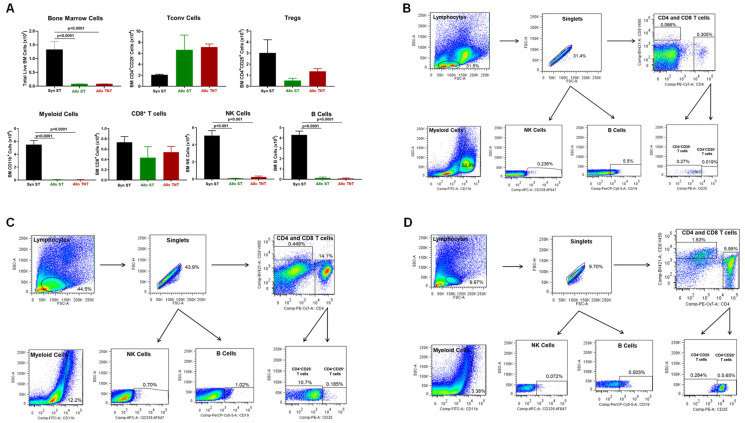
Bone marrow analysis in syngeneic and allogeneic mice housed at different temperatures. (**A**) Total BM cells and BM-residing immune cells were quantified at 4 weeks post T cell transfer for syngeneic mice and prior to euthanasia for allogeneic mice. Conventional T cells (Tconv cells; CD4^+^CD25^−^), regulatory T cells (Tregs; CD4^+^CD25^+^), Myeloid cells (CD11b^+^), CD8^+^ T cells, NK cells (CD335^+^) cells and B cells (CD19^+^) were quantified by flow cytometry as described in the Methods Section 2.4. (**B**–**D**) Representative flow cytometry plots of Syn ST mice, AlloST mice and AlloTNT mice. The number of mice in the three groups is presented in Figure 5. Data represent the mean ± SEM.

Adoptive transfer of Bl6/45.1 Tconv cells also resulted in a remarkable loss (>85%) of BM cellularity in both the AlloST and AlloTNT groups when compared to their syngeneic controls that corresponded to large and significant reductions of myeloid cells, NK cells and B cells in both groups (Figure 7).

**Figure 8:**
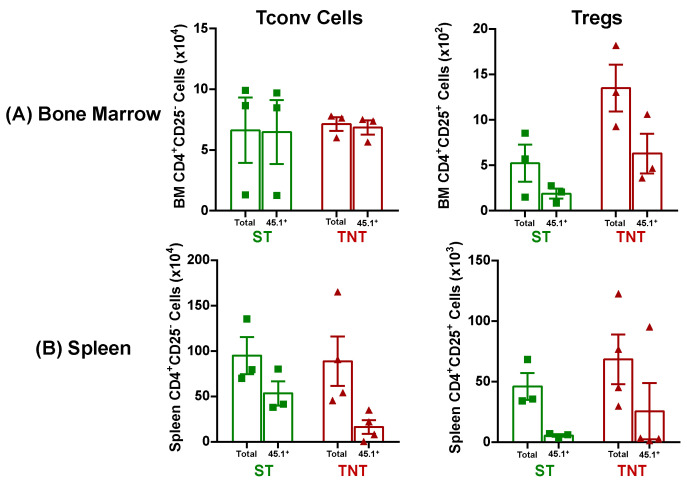
T cell engraftment in bone marrow and spleen of mice housed at different temperatures. (**A**) Total and donor-derived Tconv cells and Tregs in BM of mice housed at ST or TNT were quantified by flow cytometry. Donor (i.e., allogeneic) Tconv cells and Tregs in BM of mice housed at the two temperatures were quantified by flow cytometry using the congenic CD45.1 marker expressed on allogeneic Bl6 T cells. (**B**) Total and donor-derived Tconv cells and Tregs in the spleens of mice housed at ST or TNT were quantified as described in (**A**). The number of mice in the BM and spleen groups is N = 3 for both. Data represent the mean ± SEM.

**Figure 9:**
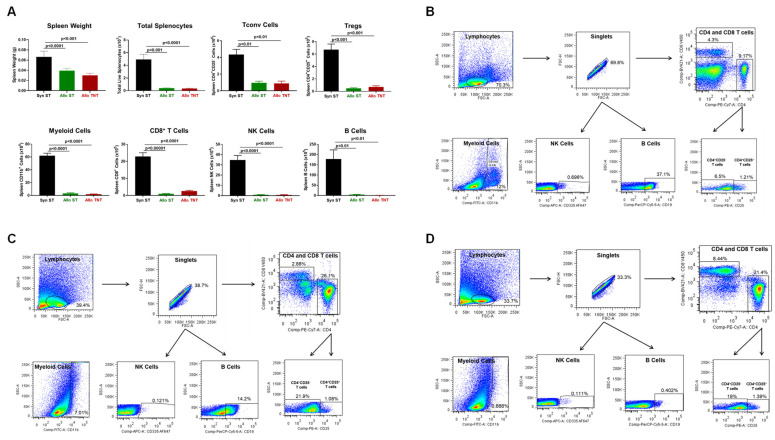
Spleen weights and immune cell analyses in syngeneic and allogeneic mice housed at different temperatures. (**A**) Spleen weights, splenocyte numbers and spleen-residing immune cells were quantified at 4 weeks post T cell transfer for syngeneic mice and prior to euthanasia for allogeneic mice. Conventional T cells (Tconv cells; CD4^+^CD25^−^), regulatory T cells (Tregs; CD4^+^CD25^+^), Myeloid cells (CD11b^+^), CD8^+^ T cells, NK cells (CD335^+^) cells and B cells (CD19^+^) were quantified by flow cytometry as described in the Methods Section 2.4. The number of mice in the three groups is presented in Figure 5. Data represent the mean ± SEM. (**B**–**D**) Representative flow cytometry plots of Syn ST mice, AlloST mice and AlloTNT.

Using the congenic CD45.1 marker expressed on allogeneic Bl6 T cells to identify donor T cells in recipient tissue, we found, using the mean T cell values, that virtually all Tconv in the BM of AlloST and AlloTNT mice were derived from Bl6/45.1 donors, whereas 40% and 48% of Treg populations in the BM were derived from the Bl6/45.1 donors in AlloST and AlloTNT mice, respectively (Figure 8A). Of note, the number of *allogeneic* Tconv cells within the BM of AlloST and AlloTNT mice was 125 and 50 times greater than the *total* number of Tregs in BM at these two temperatures, respectively (Figure 8A). Adoptive transfer of 5k/gbw Bl6/45.1 Tconv cells into sub-lethally irradiated recipients also induced substantial spleen damage that included major reductions in spleen weights in both AlloST and AlloTNT mice as well as striking losses of splenocyte numbers, Tregs, Tconv cells, myeloid cells, CD8^+^ T cells, NK cells and B cells when compared to their SynST controls (Figure 9). Using mean T cell values for the different T cell populations in the spleen, we found that 58% and 20% of all Tconv cells were derived from allogeneic donors at ST and TNT, respectively, whereas only 13% and 37% of Tregs were derived from donors at these two temperatures, respectively (Figure 8B). The mean numbers of *allogeneic* Tconv cells within the spleen in AlloST and AlloTNT mice were 10 and 1.7 times greater than the total numbers of Tregs, respectively (Figure 8B).

**Figure 10:**
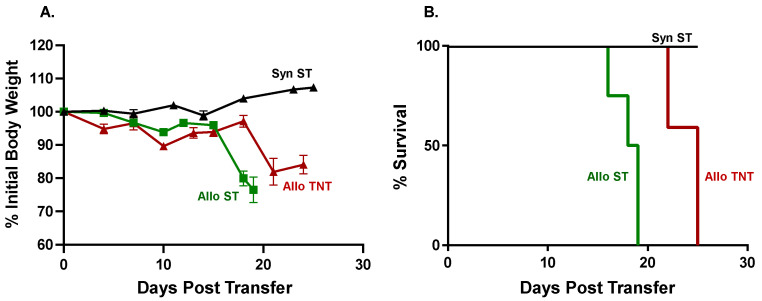
Effects of housing temperature on body weight and survival following transfer of small numbers of allogeneic T cells. Flow-sorted, allogeneic CD4^+^CD25^−^ T cells from CD45.1 Bl6 donors (2k T cells/gbw) were injected (*i.v.*) into sub-lethally irradiated BM12 recipients and housed at ST (AlloST) or TNT (AlloTNT). Sub-lethally irradiated BM12 mice injected (*i.v.*) with 2k/gbw CD4^+^CD25^−^ T cells from BM12 donors (2k/gbw) and housed at ST served as the syngeneic control group (SynST). (**A**) Body weights of mice in the three groups at different times following injection of T cells. *p* < 0.027 for SynST vs. AlloST at day 18. (**B**) Kaplan–Meir survival plots of the three groups. Mice exhibiting severe disease as evidenced by lethargy, kyphosis and/or weight loss ≥ 20% of their original weight were designated as moribund and euthanized. *p* < 0.01 for SynST vs. AlloTNT; *p* < 0.01 for SynST vs. AlloST and *p* < 0.01 for AlloST vs. AlloTNT. The starting number of mice in the SynST, AlloST and AlloTNT groups were N = 4, N = 4 and N = 5, respectively.

When taken together, these data demonstrate that allogeneic T cells traffic to the BM and spleen in RIC-treated recipients, where they induced extensive tissue damage. Although we achieved a 4-fold reduction in the number of donor Tconv cells, BM and spleen damage remained so severe at both housing temperatures that we were unable to determine whether housing temperature differentially affects the cellular composition in these two tissues. In addition, we found no significant differences in plasma concentrations of the different cytokines between the AlloST vs. AlloTNT groups. Therefore, we reduced T cell number even further by injecting (*i.v.*) 2k/gbw Bl6/45.1 Tconv cells into sub-lethally irradiated BM12 mice housed at ST or TNT. We found that engraftment with this small number of allogeneic T cells into RIC-treated recipients housed at ST or TNT resulted in the time-dependent loss of body weight and survival (Figure 10). We also observed that survival of AlloTNT mice was significantly increased by 39% when compared to their AlloST counterparts (*p* < 0.01) (Figure 10).

**Figure 11:**
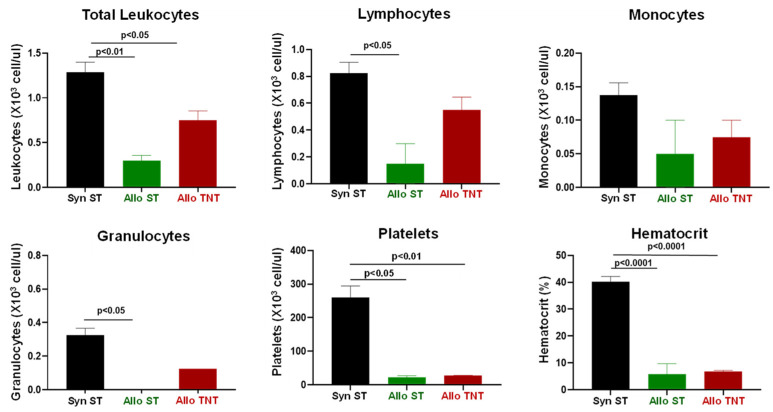
Complete blood cell counts (CBC) in syngeneic and allogeneic mice housed at different temperatures. CBC analysis was quantified from EDTA-treated whole blood from each mouse using the CBC software associated with the Heska HemaTrue Veterinary Hematology Analyzer at 4 weeks post T cell transfer for syngeneic mice and prior to euthanasia for allogeneic mice. The number of mice in the three groups is presented in Figure 10. Data represent the mean ± SEM.

CBC analysis revealed significant reductions in the total number of circulating leukocytes, platelets and hematocrit in both the AlloST and AlloTNT mice when compared to their SynST controls (Figure 11). In addition, we found that the number of circulating lymphocytes and granulocytes were selectively reduced in the AlloST but not AlloTNT group when compared to the SynST group (Figure 11).

**Figure 12:**
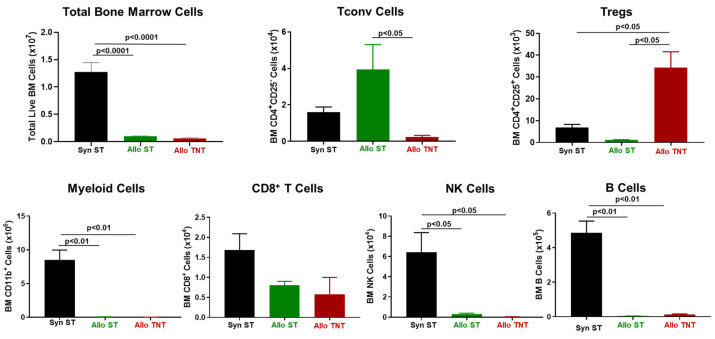
Bone marrow analysis in syngeneic and allogeneic mice housed at different temperatures. Total BM cells and BM-residing immune cells were quantified at 4 weeks post T cell transfer for syngeneic mice and prior to euthanasia for allogeneic mice. Conventional T cells (Tconv cells; CD4^+^CD25^−^), regulatory T cells (Tregs; CD4^+^CD25^+^), Myeloid cells (CD11b^+^), CD8^+^ T cells, NK cells (CD335^+^) cells and B cells (CD19^+^) were quantified by flow cytometry as described in the Methods Section 2.4 and Figure 7. The number of mice in the three groups is presented in Figure 10. Data represent the mean ± SEM.

Similar to our previous findings, we observed dramatic reductions in BM cellularity in both the AlloST and AlloTNT groups when compared to their SynST controls that correlated with large and significant decreases in myeloid cells, NK cells and B cells (Figure 12). Interestingly, we found significant increases in Tregs in the AlloTNT mice when compared to their AlloST counterparts or SynST controls, whereas Tconv cell numbers were markedly and significantly reduced in AlloTNT mice when compared to their AlloST counterparts (Figure 12).

**Figure 13:**
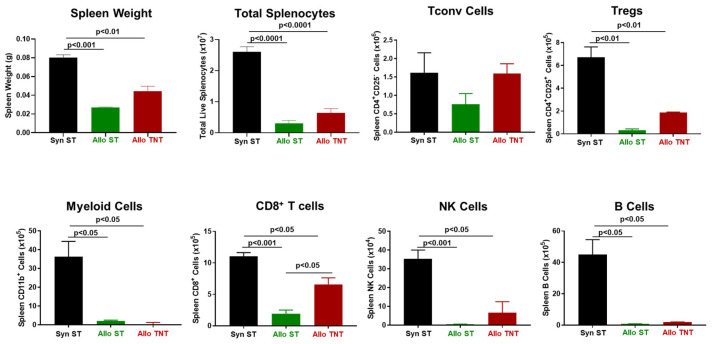
Spleen weight and immune cell analysis in syngeneic and allogeneic mice housed at different temperatures. Spleen weights, splenocyte numbers and spleen-residing immune cells were quantified at 4 weeks post T cell transfer for syngeneic mice and prior to euthanasia for allogeneic mice. Conventional T cells (Tconv cells; CD4^+^CD25^−^), regulatory T cells (Tregs; CD4^+^CD25^+^), Myeloid cells (CD11b^+^), CD8^+^ T cells, NK cells (CD335^+^) cells and B cells (CD19^+^) were quantified by flow cytometry as described in the Methods Section 2.4 and Figure 9. The number of mice in the three groups is presented in Figure 10. Data represent the mean ± SEM.

In addition, we noted significant reductions in spleen weights and marked reductions in splenocyte number, Tregs, myeloid cells, CD8^+^ T cells, NK cells and B cells in both the AlloST and AlloTNT groups when compared to their SynST controls (Figure 13). We also observed a significant increase in CD8^+^ T cell numbers in AlloTNT mice vs. the AlloST group (Figure 13).

Furthermore, we failed to observe significant differences in plasma concentrations of the different cytokines between the AlloST and AlloTNT groups. When taken together with the 5k allogeneic T cell/gbw experiments, we found that allogeneic T cells selectively damage BM and spleen in sub-lethally irradiated recipients, leading to severe anemia, BM failure and spleen hypoplasia.

### 3.3. Adoptive Transfer of Allogeneic T Cells Fails to Induce Tissue Damage in Sub-Lethally Irradiated Outbred Mice

**Figure 14:**
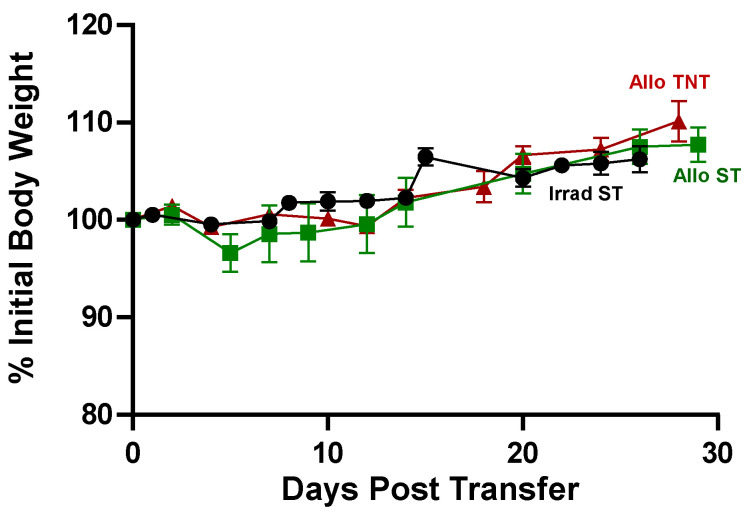
Adoptive transfer of large numbers of allogeneic CD4^+^ T cells fails to induce weight loss in *outbred* recipients. Flow-sorted, allogeneic CD4^+^CD25^−^ T cells from CD45.1 Bl6 donors (40 k T cells/gbw) were injected (*i.v.*) into sub-lethally irradiated (500 cGy) CD1 outbred recipients and housed at ST (AlloST) or TNT (AlloTNT). CD1 mice receiving irradiation alone and housed at ST (IrradST) served as the control group. The number of mice in the Irrad ST AlloST and AlloTNT groups is N = 4 for each group. Data represent the mean ± SEM.

Virtually all mouse models of aGVHD use inbred strains of mice. To our knowledge, no studies have been published using genetically diverse *outbred* mice to model aGVHD. Therefore, we initiated a series of studies to determine what effect genetic diversity has on the onset and/or severity of allogeneic T cell-induced BM and spleen damage. In a preliminary study, we injected (*i.v.*) our standard number (20k/gbw) allogeneic Bl6/45.1 (H2^b^) Tconv cells/gbw into *outbred* CD1 (*H2^b,k,p,q^*) recipients (i.e., I→O) subjected to sub-lethal irradiation (500 cGy) and housed at ST or TNT. Because CD1 mice are outbred, no two mice have identical genomes, thereby making it impossible to have a syngeneic control group. Therefore, CD1 mice that received sublethal irradiation alone but no T cells were used as our control group. Surprisingly, and in contrast to what we observed in our studies using inbred donors and recipients, we failed to observe any evidence of disease at either housing temperature as assessed by loss of body weight or alterations in CBC, BM and spleen cellularity. Therefore, we undertook a second, more detailed study in which we injected (*i.v.*) twice the number of Bl6/45.1 Tcov cells (40k/gbw) into sub-lethally irradiation CD1 recipients housed at ST or TNT to determine whether this larger number of allogeneic T cells was capable of inducing BM and spleen damage. Again, we failed to observe a loss of body weight or physical signs of disease (kyphosis, lethargy) in either group of T cell-engrafted mice (Figure 14). In fact, we found that T cell-injected CD1 recipients housed at ST or TNT experienced a 7.5% and 10% increase in weight gain, respectively, over the 30-day observation period (Figure 14).

**Figure 15:**
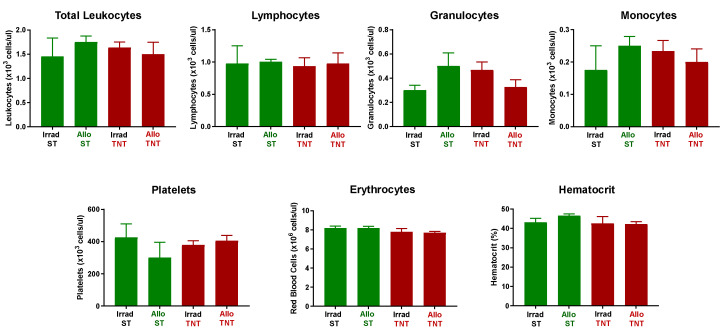
CBC analysis was quantified from EDTA-treated whole blood from each mouse using the CBC software associated with the Heska HemaTrue Veterinary Hematology Analyzer at 4 weeks post T cell transfer for all mice in the following grops: IrradST, IrradTNT, AlloST and AlloTNT. The number of mice in the IrradST, IrradTNT, AlloST and AlloTNT groups are N = 4, N = 3, N = 4 and N = 4, respectively, for each group. Data represent the mean ± SEM.

Consistent with a lack of disease, we failed to observe any major alterations in hematocrit or circulating numbers of immune cells in I→O mice at either temperature when compared to CD1 mice that received irradiation alone (Irrad) (Figure 15).

**Figure 16:**
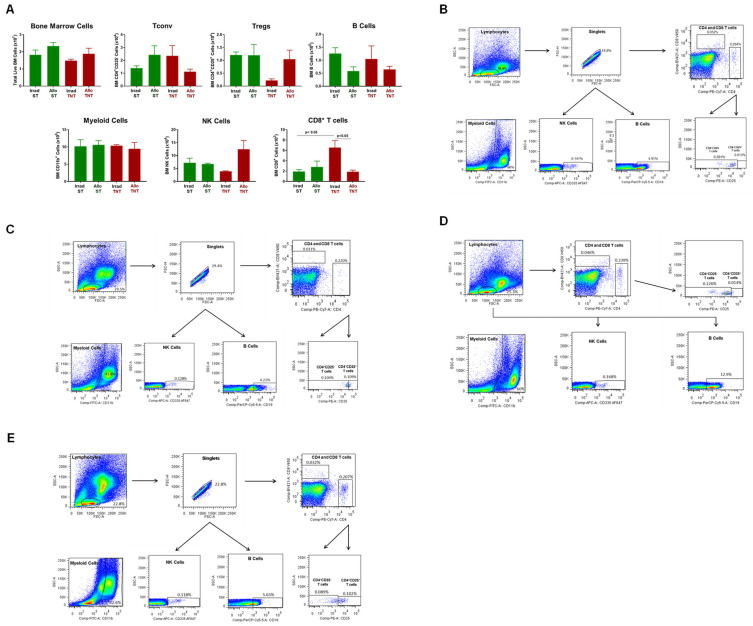
Bone marrow analysis in CD1 mice engrafted with allogeneic T cells housed at different temperatures. (**A**)Total BM cells and BM-residing immune cells were quantified at 4 weeks post T cell transfer for each mouse in the 4 groups described in Figure 15. Conventional T cells (Tconv cells; CD4^+^CD25^−^), regulatory T cells (Tregs; CD4^+^CD25^+^), Myeloid cells (CD11b^+^), CD8^+^ T cells, NK cells (CD335^+^) cells and B cells (CD19^+^) were quantified by flow cytometry as described in the Methods Section 2.4. (**B**–**E**) Representative flow cytometry plots of Irrad ST, Allo ST Irrad TNT mice and Allo TNT, respectively. The number of mice in the 4 groups are designated in Figure 15. Data represent the mean ± SEM.

**Figure 17:**
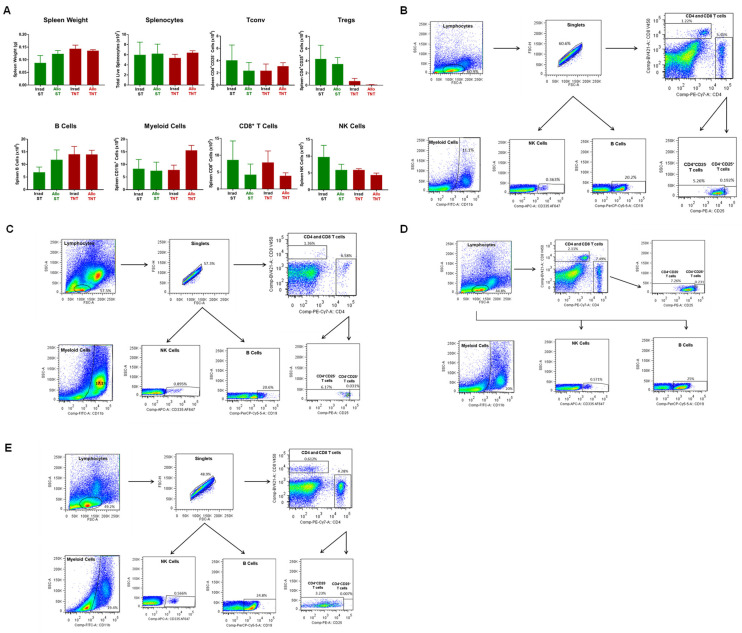
Spleen weight and immune cell analysis in CD1 mice engrafted with allogeneic T cells housed at different temperatures. (**A**) Spleen weights, splenocyte numbers and spleen-residing immune cells were quantified at 4 weeks post T cell transfer for each mouse in the 4 groups described in Figure 17. Conventional T cells (Tconv cells; CD4^+^CD25^−^), regulatory T cells (Tregs; CD4^+^CD25^+^), Myeloid cells (CD11b^+^), CD8^+^ T cells, NK cells (CD335^+^) cells and B cells (CD19^+^) were quantified by flow cytometry as described in the Methods Section 2.4. (**B**–**E**) Representative flow cytometry plots of Irrad ST, Allo ST Irrad TNT mice and Allo TNT, respectively. The number of mice in the four groups is designated in Figure 15. Data represent the mean ± SEM.

Aside from a significant reduction in BM CD8^+^ T cells in AlloTNT vs. Irrad CD1 housed at TNT, we failed to observe any other significant alterations in immune cell populations in BM and spleen (Figure 16 and Figure 17).

**Figure 18:**
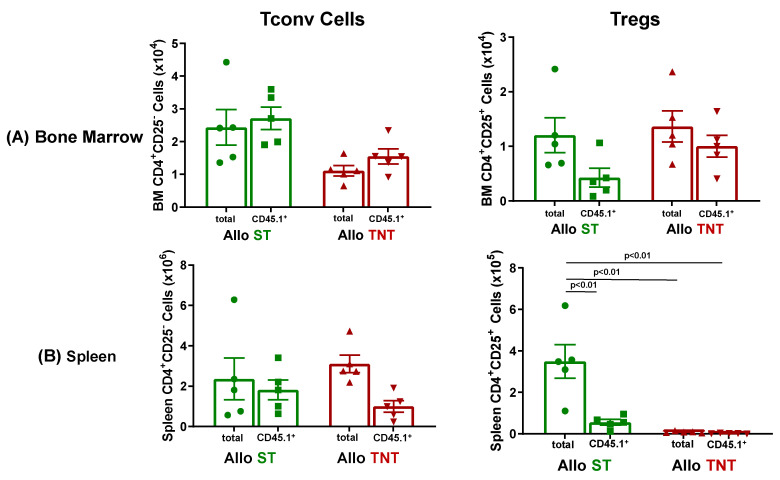
T cell engraftment in bone marrow and spleen of CD1 mice housed at different temperatures. (**A**) Total and donor-derived Tconv cells and Tregs in BM of each mice housed at ST or TNT were quantified by flow cytometry. Donor (i.e., allogeneic) Tconv cells and Tregs in BM of mice housed at the two temperatures were quantified by flow cytometry using the congenic CD45.1 marker expressed on allogeneic Bl6 T cells. (**B**) Total and donor-derived Tconv cells and Tregs in the spleens of mice housed at ST or TNT were quantified by flow cytometry. The number of mice in the BM and spleen groups is N = 5 for both. Data represent the mean ± SEM.

Having established that CD1 mice are resistant to T cell-mediated tissue damage using a protocol that induced severe disease in inbred mice, we next wished to determine whether the lack of tissue damage in outbred CD1 mice was due to defects in the trafficking of allogeneic T cells to BM and spleen. We found that the lack of BM damage in CD1 recipients was *not* due to defects in donor T cell trafficking as we observed that virtually all of the Tconv cells in the BM of CD1 mice housed at ST or TNT were derived from the allogeneic donors, whereas 38% and 74% of BM Tregs in AlloST and AlloTNT mice, respectively, were derived from donors (Figure 18A). Based upon mean T cell numbers, we calculated that the ratio of allogeneic Bl6/45.1 Tconv cells to total Tregs in the BM of AlloST and AlloTNT mice were 2:1 and 1:1, respectively (Figure 18A), whereas these ratios in BM of I→I mice (*Bl6/45.1→BM12*) mice housed at ST or TNT were 125:1 and 50:1, respectively (Figure 8A). These data suggest that the presence of Tregs in numbers that approximate those of allogeneic Tconv cells in the BM of CD1 mice may be suppressing allogeneic T cell-mediated tissue damage. Similar to BM, the lack of spleen hypoplasia *was not* due to major defects in donor T cell trafficking to the spleen, as we observed 80% of Tconv cells in the spleens of mice housed at ST were derived from allogeneic donors, whereas only 35% of Tconv cells in spleens of mice housed at TNT were derived from donors (Figure 18B) Interestingly, only 13% of splenic Tregs in AlloST mice were derived from allogeneic donors with very few donor or recipient Tregs observed in AlloTNT mice (Figure 18B). Unlike the BM, the ratios of allogeneic Tconv cells:total Tregs in the spleens of mice housed at ST or TNT were 5:1 and >200:1, respectively, suggesting that Tregs may *not* play a role in suppressing spleen damage.

The immunological mechanisms responsible for protecting CD1 mice from allogeneic T cell-mediated damage are not apparent at the present time. However, the fact that BM and spleens in inbred Bl6/45.1→BM12 mice are essentially devoid of B cells and myeloid cells (Figure 7, Figure 9, Figure 12 and Figure 13) suggests that these immune cell populations may contain one or more subsets of immuno-suppressive cells, such as regulatory B cells (Bregs) and/or myeloid-derived suppressor cells (MDSCs) that could be mediating disease suppression. It is well known that the numbers and immunosuppressive activities of Bregs and MDSCs are induced in different mouse models of autoimmune disease, allograft rejection and inflammatory diseases [46,47,48,49,50,51,52,53,54,55,56,57]. Bregs may suppress expansion of pathogenic T cells via their production of IL-10, IL-35 and/or transforming growth factor-β (TGFβ) [55,56,58,59,60], whereas MDSCs have been shown to mediate immune suppression via the production/expression of reactive oxygen species (ROS), prostaglandin E_2_, nitric oxide (NO), IL-10, TGFβ, arginase-1 and programmed death-ligand 1 (PD-L1) [55,56]. Although we did not quantify Bregs and MDSCs in the current study, the published frequencies of these immunoregulatory cells in BM and spleen of mice [48,49,53], suggest that their numbers may approximate or even exceed the numbers of allogeneic T cells within the BM and spleen of CD1 recipients. An important yet unexplored aspect of this potential mechanism for suppressing disease is whether Breg and MDSC numbers may be altered at different housing temperatures.

An additional, but highly unlikely explanation for the lack of T cell-mediated tissue damage in Bl6/45.1→CD1 mice may be that the *H2^b^* haplotype shared by Bl6/45.1 and CD1 (*H2^b,k,p,q^*) mice may somehow limit the ability of these T cells to induce robust disease in CD1 recipients. To address this possibility, we undertook an additional study to determine whether *i.v.* administration of similar numbers of BM12 T cells (40k T cells/gbw) into sub-lethally irradiated CD1 recipients would induce disease in mice housed at ST. The MHC haplotype of BM12 mice (*H2^BM12^*) is a *complete mismatch* with CD1 mice. We found that the adoptive transfer of BM12 T cells into sub-lethally irradiated CD1 mice failed to alter the circulating numbers of the different immune cell populations and hematocrit (Figure S8), nor did it alter many of the different BM and spleen immune cell populations when compared to their irradiated controls (Figures S9 and S10). We did observe significant reductions in total BM cells and Tregs in mice injected with BM12 T cells (Figure S9); however, we did not observe any significant alterations in spleen immune cell populations (Figure S10). When taken together, these data demonstrate that *i.v.* administration of large numbers of two different populations of allogeneic T cells into sub-lethally irradiated CD1 mice is incapable of inducing BM and spleen damage in mice housed at ST.

## 4. Discussion

The vast majority of mouse models of GVHD use inbred mice that are housed at ST. Although these studies have provided new and promising data regarding the immuno-pathological mechanisms responsible for aGVHD, the translation of these data into more effective therapeutics for treatment of patients has been rather poor [17,18,19,20]. Although the reasons for this dismal bench-to-bedside transition are not completely understood, it is known that immune responses in mice housed at ST (22–24 °C) may be quite different than mice housed at their TNT (30–32 °C). Indeed, it has been well-documented that ST creates mild but *chronic* cold stress that can alter disease pathogenesis in mouse models of chronic disease, including aGVHD when compared with mice housed at TNT [29,30,31,32,61,62]. In addition to housing temperature, all mouse models of aGVHD use inbred strains of mice. Unfortunately, it is not clear whether immune responses of inbred mice engrafted with allogenic T cells recapitulate the diverse immune responses found in humans. In fact, several studies have found that specific immune responses and disease phenotypes may be quite different in inbred vs. genetically diverse *outbred* mice [33,34,35,36,37,38]. In an attempt to address how these differences may affect disease pathogenesis, we investigated how housing temperature and genetic diversity affect disease onset and/or severity in our model of aGVHD.

Data presented in the current study demonstrates that adoptive transfer of small numbers of allogeneic Tconv cells from inbred donors into RIC-treated inbred recipients (I→I) housed at ST or TNT selectively damages the BM and spleen showing that allogeneic Tconv cells are both necessary and sufficient to induce tissue-specific damage to these hematopoietic tissues. Our data show that the survival of T cell-engrafted mice housed at TNT is significantly greater when compared to their counterparts housed at ST. In addition, we demonstrate, for the first time, that adoptive transfer of allogeneic Tconv cells into RIC-treated *outbred* mice failed to induce disease in mice housed at ST or TNT, suggesting MHC-independent mechanisms of resistance to T cell-mediated BM and spleen damage. To our knowledge, our study is the first to report how housing temperature and genetic diversity may influence the development and severity of tissue damage in this model of aGVHD.

### 4.1. T Cell Trafficking and Tissue Damage

A unique aspect of the RIC-treated inbred mouse model used in the current study is that allogeneic T cell-mediated BM and spleen damage occurs in the absence of injury to those tissues (i.e., gut, liver and skin) that are known to develop in conventional, lethally-irradiated mouse models of aGVHD (Figure S1). In fact, variations of the model described in our current study have been used by other investigators to model immune-mediated aplastic anemia via adoptive transfer of unfractionated, allogeneic lymph node or spleen cells into sub-lethally irradiated recipients [7,63,64,65]. Our work extends the work of these investigators by demonstrating that flow-purified CD4^+^ Tconv cells are both necessary and sufficient to selectively induce BM and spleen hypoplasia in RIC-treated recipients. Together, these data suggest tissue-specific trafficking of allogeneic Tconv cells in recipients exposed to RIC. It is well known that naïve and certain effector T cells express the chemokine receptor CXCR4 that is required for their homing to BM and spleen [8,63]. Using a *conventional* model of aGVHD, Chewning et al. reported that injection of naïve allogeneic CD4^+^ T cells together with donor BM cells into *lethally* irradiated recipients induced inflammatory tissue damage in multiple organs, including the gut, lung, skin, BM and spleen [8]. When these investigators transferred in vivo- or in vitro-generated allogeneic Th1 cells together with donor BM cells into lethally-irradiated recipients, they observed only BM and spleen damage that was dependent upon T cell expression of CXCR4 [8]. The reasons why these effector T cells only traffic to these two tissues in mice that receive lethal, total body irradiation are not clear but are most likely due to the selective expression of the CXCR4 chemokine ligand CXCL12 by BM and spleen stromal cells [8,63,66]. In addition, Chewning et al. noted that Th1 cells lack additional homing chemokine receptors (e.g., CCR6 and CCR9) that may be used to traffic to other tissues [8]. The mechanisms by which allogeneic Tconv cells mediate tissue damage were not defined in the present study but most likely involve Th1 and possibly Th17 effector cells. Th1 effector cells may mediate cell and tissue damage by induction of apoptosis of hematopoietic progenitor cells via their production of TNFα and IFNγ as well as FAS–FAS ligand interactions [3,8,16]. The role of Th17 effector T cells in the pathogenesis of aGVHD is controversial as some investigators have reported that these effector T cells are sufficient to drive disease, whereas others have reported that Th17 cells may actually be protective in mouse models of aGVHD [67,68]. Lack of disease in the typical target tissue (gut, liver, skin) in mice subjected to RIC likely reflects reduced expression of additional chemokine ligands on endothelial cells within these tissues. Nonspecific tissue damage produced by myeloablative, total body irradiation conditioning protocols is known to create a systemic inflammatory milieu that induces the expression of different T cell-associated chemokine receptors (e.g., CXCR3, CCR2, CCR5) and chemokine ligands (CXCL9, CXCL10, CXCL11) in the multiple target tissues that direct the homing of T cells to the different organ systems [24,69]. It should also be noted that data generated in the current study may have important clinical implications given the fact that many patients are currently being treated with RIC protocols prior to HSCT to reduce the development of multiorgan aGVHD. Our data suggest that while RIC may significantly reduce disease in many of the major target organs, hematopoietic tissue damage persists, rendering recipients susceptible to recurrent infections and bacteremia.

### 4.2. Effect of Housing Temperature on Survival and Tissue Damage

Another important finding reported in the current study is that housing temperature has an effect on the survival of RIC-treated mice engrafted with allogeneic Tconv cells. Surprisingly and in contrast to reports by others using the conventional, *lethally*-irradiated mouse model of aGVHD [61,62], we found that survival of RIC-treated mice injected with small numbers of allogeneic Tconv cells housed at TNT was significantly *increased* when compared to T cell-engrafted mice housed at ST (Figure 7 and Figure 12). Leigh et al. reported that when inbred mice were subjected to lethal irradiation and injected with allogeneic BM and T cells, 100% of the mice housed at ST survived the 60-day observation period, whereas only 50% of the mice housed at TNT survived this same time period [61]. These investigators determined that ST housing protects mice from the deleterious effects of aGVHD by increasing β-adrenergic receptor signaling in immune cells. They found that when mice were housed at ST and then treated with a pan-β blocker (i.e., propranolol), the severity of disease increased, suggesting that β-adrenergic receptor signaling is protective against aGVHD [61,62]. The reasons for differences in survival at the two housing temperatures in our studies vs. those described above using the conventional model of aGVHD are not apparent; however, they may be related to the differences in pretransplant conditioning protocols (lethal vs. sublethal conditioning) and/or the use of flow-sorted Tconv cells vs. unfractionated T cells together with donor BM.

A question that emerges from our housing temperature studies is why ST housing reduces the survival of I→I mice when compared to their TNT counterparts, given that both groups of mice exhibited similar degrees of severe anemia and thrombocytopenia as well as similar decrements in BM and spleen cellularity. We originally hypothesized that reduced survival in mice housed at ST may be due to a paucity of Tregs relative to the numbers of allogeneic Tconv cells in the BM when compared to mice housed at TNT. Although we did observe that the ratio of allogeneic Tconv cells:total Tregs in BM of ST mice was 2.5 times greater than that of TNT mice (125:1 vs. 50:1, respectively), the literature suggests that at these ratios, Tregs would exert little or no immunosuppressive effect at either temperature (Figure 10A) [70]. It is also possible that the reduced survival of mice housed at ST may be due to increased circulating levels of inflammatory cytokines (e.g., TNFα, IFN-γ, IL-6 etc) in AlloST vs. AlloTNT mice. However, we failed to observe significant increases in any of the inflammatory cytokines in the plasma of AlloST vs. AlloTNT mice. Using a mouse model of sepsis, Carpenter et al. reported that the survival of *inbred* mice housed at their TNT was significantly *increased* compared to their ST counterparts [71]. They found that increased survival at TNT corresponded to reductions in bacterial cell numbers together with increased neutrophilic phagocytic activity within the peritoneal cavity. Although we did not culture the blood or peritoneal fluid in the current study, it is possible that the immune deficiency created in our mice would likely result in bacterial translocation and expansion, resulting in bacteremia. If this is the case, TNT housing may enhance the phagocytic activity of the remaining phagocytic leukocytes, thereby extending survival in these mice. In addition to housing temperature, other environmental factors may significantly influence the onset and severity of other auto- and alloimmune diseases [20]. Li et al. reported, using a similar model to the one described in the current study, that BM damage was significantly reduced in mice that were housed at ST under conventional conditions (CC) when compared to mice housed at ST in a standard barrier facility under specific pathogen-free (SPF) conditions [72]. Attenuated disease was associated with greater intestinal bacterial diversity in CC mice vs. that observed in the SPF group. It may be that housing mice at their TNT alters their microbial composition to one that enhances animal survival. As pointed out previously, the large majority of mouse studies use ST and SPF conditions [20]. These data suggest that maintaining bacterial diversity may prove beneficial to patients undergoing HSCT [72].

### 4.3. Outbred Mice Are Resistant to T Cell-Mediated Tissue Damage

Another interesting and particularly novel finding made in the current study was the inability of allogeneic T cells to induce BM and spleen damage in RIC-treated *outbred* mice housed at ST or TNT (Figure 15, Figure 16 and Figure 17 and Figures S7–S9). As pointed out previously, all mouse models of aGVHD use inbred donors and recipients. These genetically-constrained mice are generated by brother/sister mating for at least 20 generations producing genetically-identical animals. While current mouse models of aGVHD have been critical to our understanding of many of the immunopathological mechanisms involved in this disease, they most likely do not encapsulate the hybrid vigor and genetic diversity present in humans. To our knowledge, data reported in the current study are the first to evaluate the susceptibility of outbred mice in developing aGVHD. Our data demonstrate that MHC II disparity alone does not drive T cell-mediated tissue damage, suggesting that uncharacterized immune mechanisms may contribute to disease resistance. Differences in susceptibility and/or resistance to autoimmune diseases have been well-described in mouse models of autoimmune encephalomyelitis and diabetes [66,73]. We hypothesized that resistance of RIC-treated CD1 mice to T cell-mediated tissue damage may be due to the presence of large numbers of Tregs relative to the numbers of allogeneic Tconv cells in BM and/or spleen. We did observe that the total numbers of Tregs approximated those of allogeneic Tconv cells in BM of mice housed at ST and TNT (Tconv cells:total Tregs were 2:1 and 1:1, respectively) (Figure 18), suggesting that expansion and/or activation of Tregs may play an important role in protecting the BM. However, using this same analysis for the spleen, we found that the ratios of allogeneic Tconv cells:total Tregs in the spleens were 5:1 and >200:1 in mice housed at ST or TNT, respectively, suggesting that Tregs are unlikely to play a role in protecting the spleen (Figure 18).

### 4.4. Potential Mechanism Involved in Suppressing Disease in Outbred Mice

A striking feature of the I→I models described in the current study is the remarkable loss of BM and spleen B cells and myeloid cells that does *not* occur in the I→O models. These two immune cell populations are known to contain subsets of cells with potent immunoregulatory properties, such as Breg cells and MDSCs [55,58]. B10 cells are the major IL-10-producing Breg and are found within immature B cells, mature B cells, plasmablasts and plasma cells [48,49,58,59,74,75]. It is well known that the numbers and suppressive activities of B10 cells are increased in response to inflammation ([58] and references therein). Preclinical studies report that the administration of ex vivo-activated Bregs is effective at attenuating tissue damage observed in mouse models of GVHD [47,50,51,56,58]. The large decrement of BM and spleen B cells observed in our I→I models is also observed in patients with GVHD [47,50,56,58]. Clinical studies have shown that the presence of large numbers of donor-derived B cell progenitors in recipient BM is *inversely* correlated with the development of GVHD [76,77]. Additional patient-based studies have shown that expansion of B cell progenitors following HSCT was associated with less severe GVHD [78,79]. Of note, patients with severe, T cell-mediated BM failure (e.g., immune-mediated aplastic anemia) have much lower numbers of BM and circulating Bregs when compared to healthy controls [80,81]. Although did not quantify B10 cells, we estimated, based upon published frequencies of these cells in the BM and spleen of mice [48,49,53], that Bregs may approximate or exceed the numbers of allogeneic T cells within the BM and spleen of CD1 recipients. In some respects, our data are similar to those reported by Marin et.al. who showed that while Bl6 mice were susceptible to induction of experimental allergic encephalitis (EAE), CD1 mice were resistant [66]. They reported that resistance to disease induction in CD1 mice corresponded to significant increases in the numbers of Bregs and Tregs following their immunization with the disease-producing MOG_35–55_ peptide when compared to immunized Bl6 mice [66].

The other population of immunosuppressive immune cells that may confer resistance to allogeneic T cell-mediated damage are MDSCs. These cells are a heterogeneous population of immature myeloid cells that have been shown to be greatly increased during different pathological conditions, such as chronic infection and inflammation, trauma and cancer, as well as autoimmune and alloimmune diseases [53,54,55,56]. The two major groups of MDSCs identified in mice and humans are polymorphonuclear (PMN) MDSCs (CD11b^+^Ly-6G^+^Ly-6C^low^) and monocytic (M) MDSCs (CD11b^+^Ly-6G^−^Ly-6C^high^) [53,55]. Because there are no specific cell surface markers that definitively differentiate PMN-MDSCs M-MDSCs from mature PMNs and monocytes, respectively, the two MDSC populations are defined by their ability to suppress different immune responses mediated by T cells, B cells and NK cells in vitro [55]. We estimated, based upon published frequencies of these cells in the BM and spleen of mice [48,49,53], that these myeloid cells may approximate or exceed the numbers of allogeneic T cells within the BM and spleen. Feng et al. have recently reported that PMN-MDSCs potently suppress T cell-mediated BM failure using a mouse model of immune-mediated aplastic anemia [82]. Conceptionally, these data are similar to those of Dong et al. who reported that patients with severe immune-mediated aplastic anemia have fewer circulating M-MDSCs that possess impaired immunosuppressive activity [83].

## 5. Conclusions

Data presented in the current study demonstrate that adoptive transfer of small numbers of allogeneic CD4^+^ Tconv cells from inbred donors into RIC-treated inbred recipients (I→I) selectively traffic to and damage the BM and spleen. Housing mice at their TNT was found to significantly increase their survival when compared to their ST counterparts. These novel findings suggest that while RIC markedly reduces inflammation in several other target organs that are known to develop disease in mice subjected to lethal/myeloablative pretransplant conditioning, BM and spleen damage may persist, rendering mice (and possibly RIC-treated patients) susceptible to recurrent infections and bacteremia. Surprisingly, and in contrast to what we observed in I→I mice, we report, for the first time, that RIC-treated outbred mice are resistant to allogeneic T cell-mediated BM failure and spleen hypoplasia. Lack of disease in these outbred mice is associated with the presence of large numbers of B cells and myeloid cells that are known to contain immunosuppressive Breg cells and MDSCs.

## Figures and Tables

**Table 1:** Plasma cytokine concentrations in Bl6 mice engrafted with syngeneic or allogeneic CD4^+^CD25^−^ T Cells. RIC-treated Bl6 were injected (*i.p.*) with 20k/gbw Bl6 T cells (Syngeneic) or BM12 T cells (Allogeneic) and housed at standard temperature. Plasma cytokine concentrations were quantified by flow cytometry as described in the Methods section. The number of mice in the syngeneic and allogeneic groups are N = 4 and N = 3, respectively. Data represent the mean ± SEM.

Cytokine	Syngeneic	Allogeneic	*p* Value
GM-CSF	18.6 ± 18.0	0	0.4366
IFN-β	0	0	------
IFN-γ	0	15.67 ± 3.3	0.0024
IL-10	109 ± 20	141 ± 33	0.416
IL-12p70	0	0	------
IL-17A	3.29 ± 3.30	0	0.1692
IL-1α	28.4 ± 13.0	4.21 ± 2.30	0.1801
IL-1β	19.4 ± 2.63	21.0 ± 1.80	0.6492
IL-23	0	0	------
IL-27	1130 ± 582	299 ± 113	0.2851
IL-6	0	137± 99	0.1575
MCP-1	13.9 ± 2.0	107 ± 48	0.0691
TNF-α	1.26 ± 3.3	49.4 ± 14.3	0.0123

## Data Availability

The datasets used and/or analyzed for the current study are available from the corresponding author upon reasonable request.

## Supplementary Materials

The following supporting information can be downloaded at: https://www.mdpi.com/article/10.3390/pathophysiology30040039/s1. Figure S1: Adoptive transfer of allogeneic CD4+ T cells from Bl6 donors into BM12 recipients induces lethal GVHD; Figure S2: Complete blood cell counts (CBC) in syngeneic and allogeneic mice; Figure S3: Bone marrow analysis in syngeneic and allogeneic mice; Figure S4: Allogeneic CD4+ T cells induce bone marrow failure and spleen aplasia; Figure S5: Spleen weight and immune cell analysis in syngeneic and allogeneic mice; Figure S6: Complete blood cell counts (CBC) of CD1 outbred mice engrafted with BM12 T cells housed at standard temperature; Figure S7: Bone marrow analysis in CD1 mice engrafted with BM12 T cells housed at standard temperature; Figure S8: Spleen weight and immune cell analysis in CD1 mice engrafted with BM12 T cells housed at standard temperature. Figure S9: Bone marrow analysis in CD1 mice engrafted with BM12 T cells housed at standard temperature. Figure S10: Spleen weight and immune cell analysis in CD1 mice engrafted with BM12 T cells housed at standard temperature.
