# Supplementary material for: Influence of Housing Temperature and Genetic Diversity on Allogeneic T Cell-Induced Tissue Damage in Mice
Source: Pathophysiology. 2023 Nov 20;30(4):522–47. doi: 10.3390/pathophysiology30040039 (PMC10661280; doi:10.3390/pathophysiology30040039)
Supplement: Supplementary file 1 [file pathophysiology-30-00039-s001.zip › pathophysiology-2653380-supplementary.pdf]

Supplementary Figures

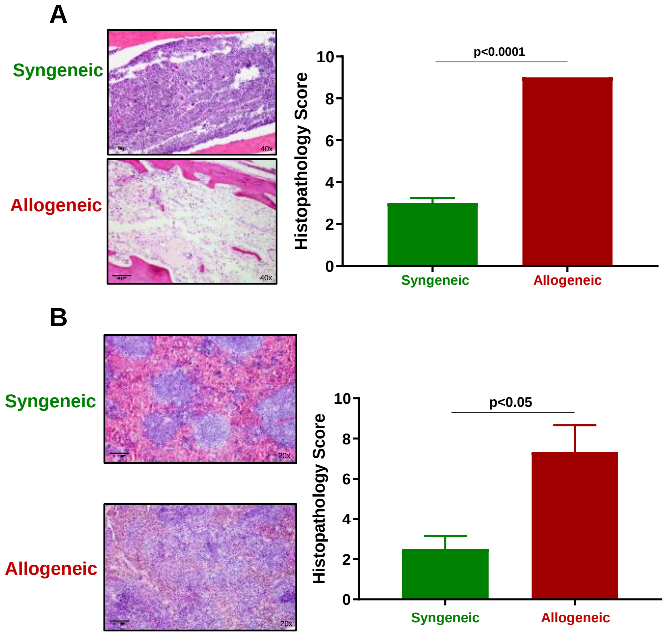

Figure S1. Histopathological analysis of bone marrow and spleen in syngeneic and allogeneic mice. (A) Histopathological alterations of bone marrow (BM) obtained from each mouse in the two groups described in Figure 1 upon euthanasia. Left hand micrographs show representative images of syngeneic and allogeneic BM (40x total magnification) with right hand graphs showing blinded histopathology scores of BM from syngeneic and allogeneic mice. Remarkable BM aplasia with little or no hematopoietic tissue is present in allogeneic BM. The number of mice in the syngeneic and allogeneic groups are N=3 and N=4, respectively. Data represent the mean ± SEM. **(B)** Histopathological alterations of spleens obtained from each mouse in the two groups described in Figure 1. Left hand micrographs show representative images of syngeneic and allogeneic spleens (40x total magnification) with right hand graphs showing blinded histopathology scores of spleens from syngeneic and allogeneic mice. Notable loss of white pulp and immune cells as well as major disruptions in the red pulp are observed in allogeneic spleens. The number of mice in the syngeneic and allogeneic groups are N=4 each. Data represent the mean ± SEM.

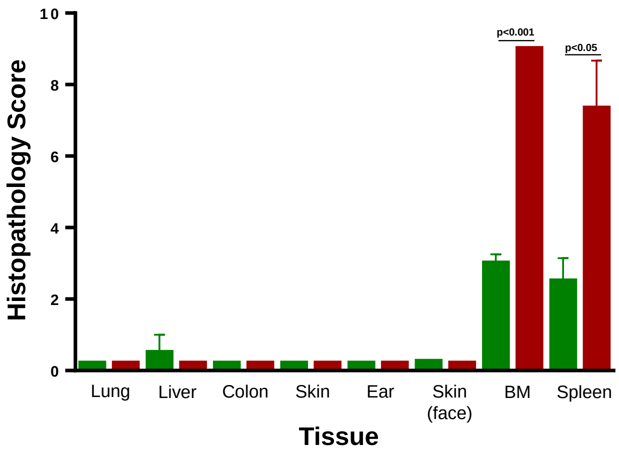

Figure S2. Histopathological analysis of different tissues from syngeneic and allogeneic mice. Blinded histopathology scores of each tissue were quantified for each mouse in the 2 groups described in Figure 1. The number of mice in the syngeneic and allogeneic groups are N=4 each. Data represent the mean ± SEM.

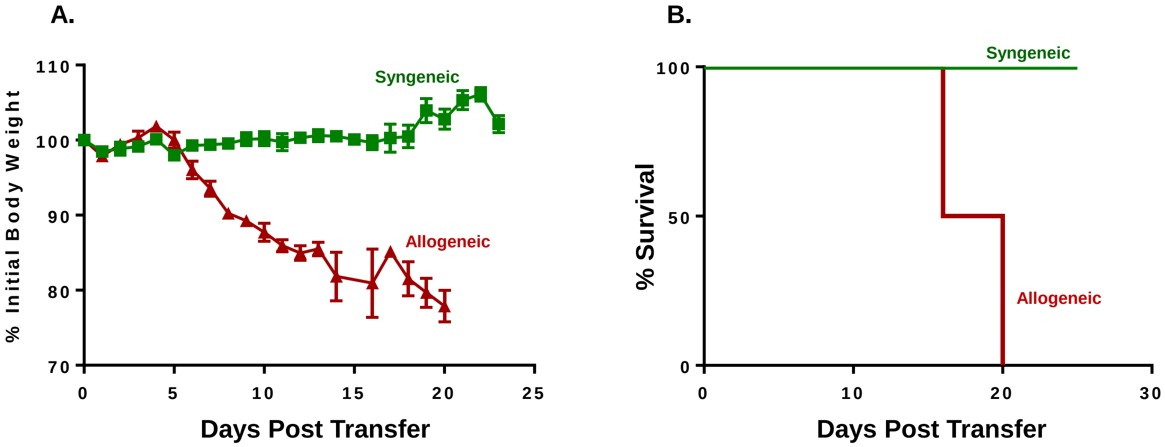

Figure S3. Adoptive transfer of allogeneic CD4$^+$ T cells from Bl6 donors into BM12 recipients induces lethal GVHD. Flow sorted, CD4$^+$CD25$^-$ T cells from syngeneic BM12 donors (20k T cells/gbw) or allogeneic T cells from Bl6 mice (20k T cells/gbw) were injected (*i.p.*) into sub-lethally irradiated BM12 recipients as described in Figure 1. **(A)** Body weights of mice at different times following injection of T cells. Data presented represent the mean ± SEM. Syngeneic vs. Allogeneic is $p<0.009$. **(B)** Kaplan-Meir survival curves. Mice exhibiting severe disease as evidenced by lethargy, kyphosis (hunched appearance) and/or weight loss ≥ 20% of their original weight were designated as moribund and euthanized. Syngeneic vs. Allogeneic is $p<0.001$. The starting number of mice in the syngeneic and allogeneic groups are N=4 for each.

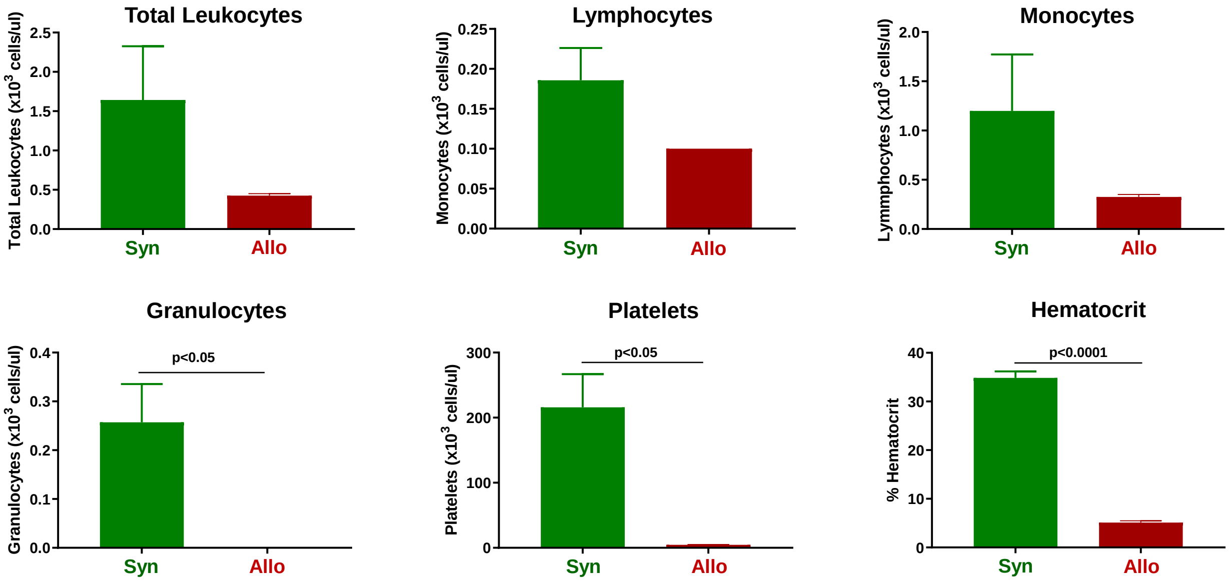

Figure S4. Complete blood cell counts (CBC) in syngeneic and allogeneic mice. CBC analysis was quantified from EDTA-treated whole blood from each mouse using the CBC software associated with the Heska HemaTrue Veterinary Hematology Analyzer at 4 weeks post T cell transfer for syngeneic mice and prior to euthanasia for allogeneic mice. The number of mice in the syngeneic and allogeneic groups are N=7 and N-4, respectively. Data represent the mean ± SEM.

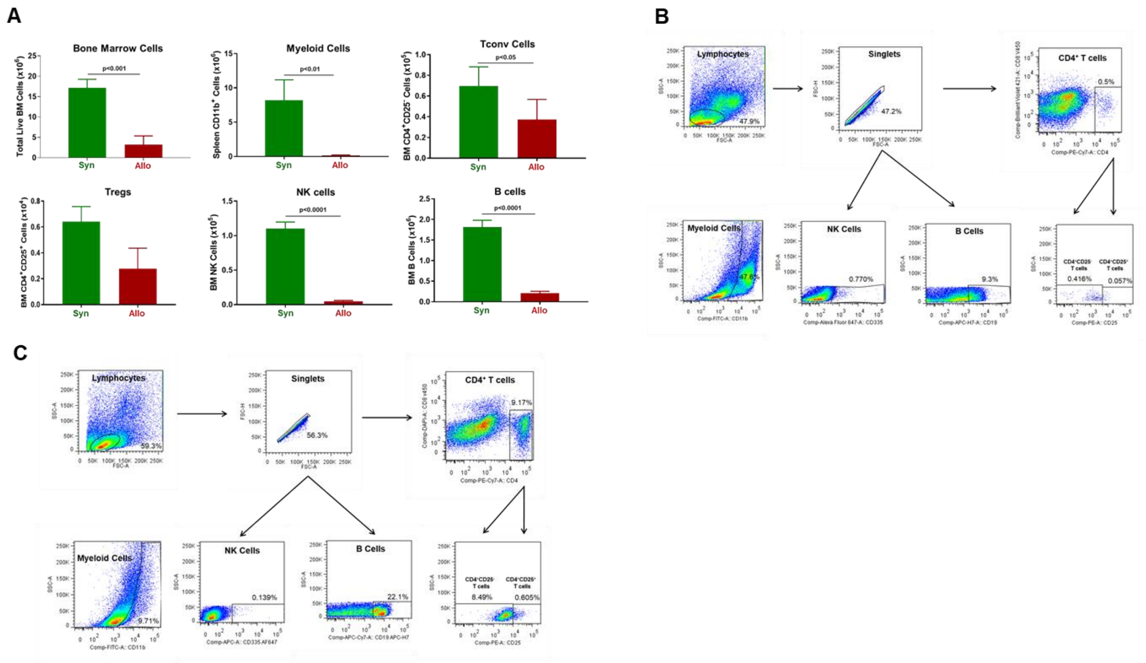

Figure S5. Bone marrow analysis in syngeneic and allogeneic mice. (A) Total BM cells as well as BM-residing immune cells were quantified at 4 weeks post T cell transfer for syngeneic mice or prior to euthanasia for allogeneic mice for each mouse in the 2 groups described in Figure S3. Myeloid cell (CD11b), Conventional T cells (Tconv cells; $CD4^+CD25^-$), regulatory T cells (Tregs; $CD4^+CD25^+$), NK cells ($CD335^+$) cells and B cells ($CD19^+$) were quantified by flow cytometry

as described in the Methods section 2.4. **(B)** and **(C)** Representative flow cytometry plots of syngeneic mice and allogeneic mice, respectively. The number of mice in the syngeneic and allogeneic groups are N=3 and N=6, respectively. Data represent the mean ± SEM.

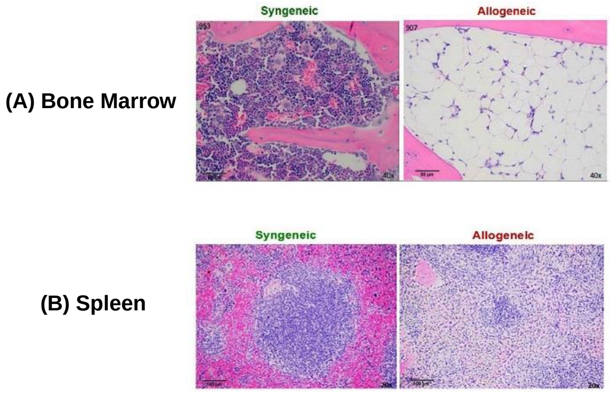

Figure S6. Allogeneic CD4+ T cells induce bone marrow failure and spleen aplasia. Representative histological images of bone marrow and spleen obtained from an untreated BM12 mouse as well as from a syngeneic and allogeneic mouse at 4 weeks post T cell transfer. **(A)** Note the remarkable aplasia in BM from mice engrafted with allogeneic T cells. **(B)** Remarkable loss of white pulp and its associated immune cells as well as red pulp in spleens of mice engrafted with allogeneic T cells.

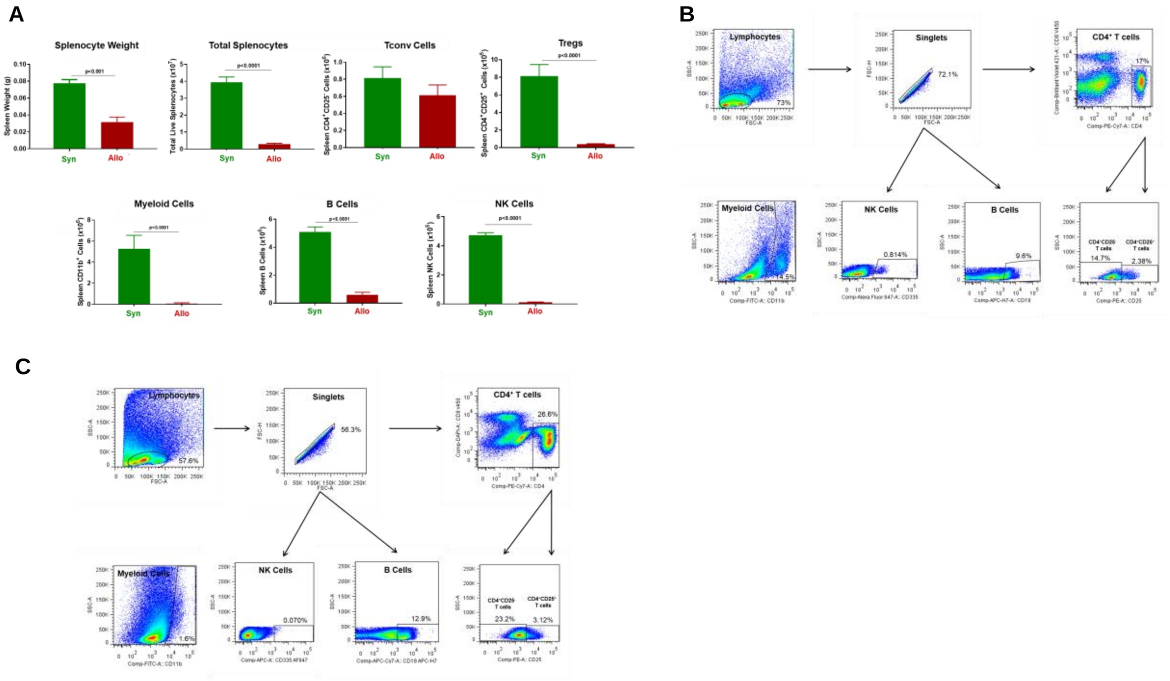

Figure S7. Spleen weight and immune cell analysis in syngeneic and allogeneic mice. (A) Spleen weights, splenocyte numbers and spleen-residing immune cells were quantified at 4 weeks post T cell transfer for syngeneic mice or prior to euthanasia for allogeneic mice. Conventional T cells (Tconv cells; $CD4^{+}CD25^{-}$), regulatory T cells (Tregs; $CD4^{+}CD25^{+}$), Myeloid cells ($CD11b^{+}$), B cells ($CD19^{+}$) and NK cells ($CD335^{+}$) cells were quantified by flow cytometry as described in the Methods section 2.4. **(B)** and **(C)** Representative flow cytometry plots of syngeneic mice and allogeneic mice. The number of mice in the syngeneic and allogeneic groups are N=3 and N=6, respectively. Data represent the mean ± SEM.

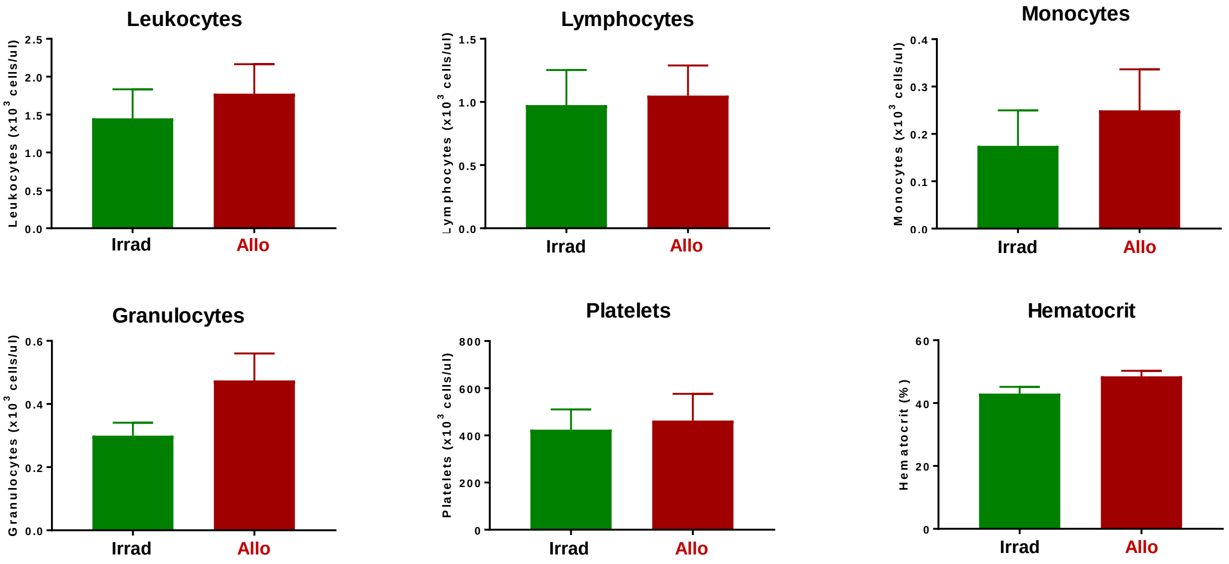

Figure S8. Complete blood cell counts (CBC) of CD1 outbred mice engrafted with BM12 T cells housed at standard temperature. CBC analysis was quantified from EDTA-treated whole blood obtained from each mouse at 4 weeks post transfer from the irradiation alone (Irrad; 500cGy) and the allogeneic (Allo) groups using the CBC software associated with the Heska HemaTrue Veterinary Hematology Analyzer. The number of mice in the Irrad and Allo groups are N=4 for each. Data represent the mean ± SEM.

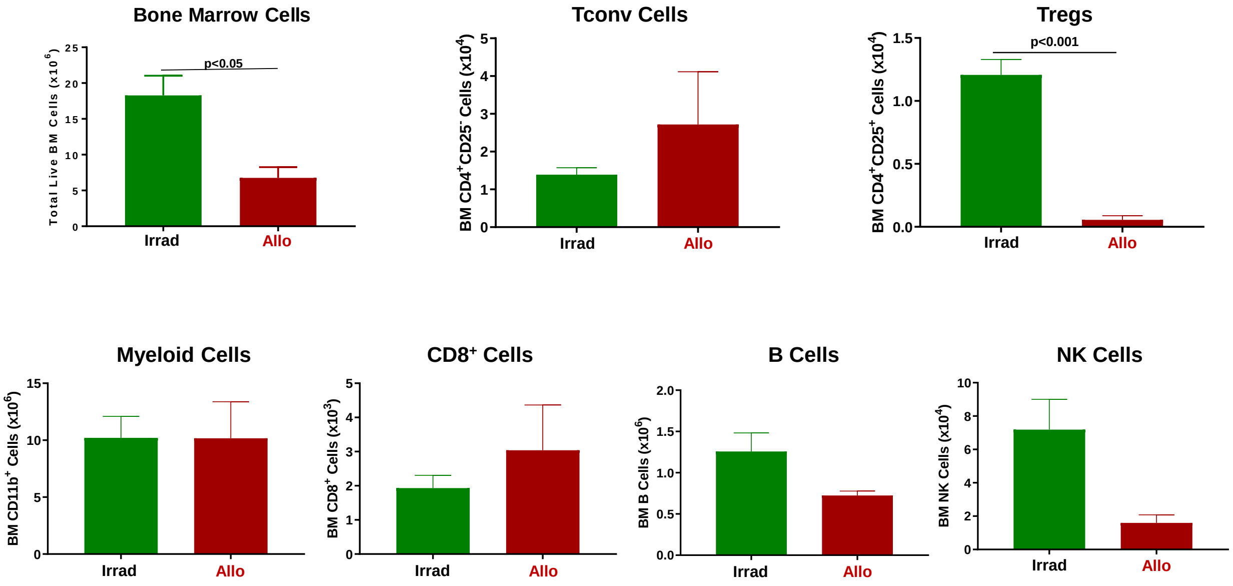

Figure S9. Bone marrow analysis in CD1 mice engrafted with BM12 T cells housed at standard temperature. (A) Total BM cells as well as BM-residing immune cells were quantified at 4 weeks post T cell transfer for each mouse in the 2 groups described in Figure S6. Conventional T cells (Tconv cells; $CD4^+CD25^-$), regulatory T cells (Tregs; $CD4^+CD25^+$), Myeloid cells ($CD11b^+$), CD8 T cells ($CD8^+$), B cells ($CD19^+$) and NK (335^+) cells were quantified by flow cytometry as described in the Results (section 2.4). **(B)** Representative flow cytometry plot of syngeneic mice and **(C)**

representative flow cytometry plot of allogeneic mice. The number of mice in the 2 groups are described in Figure S8. Data represent the mean ± SEM.

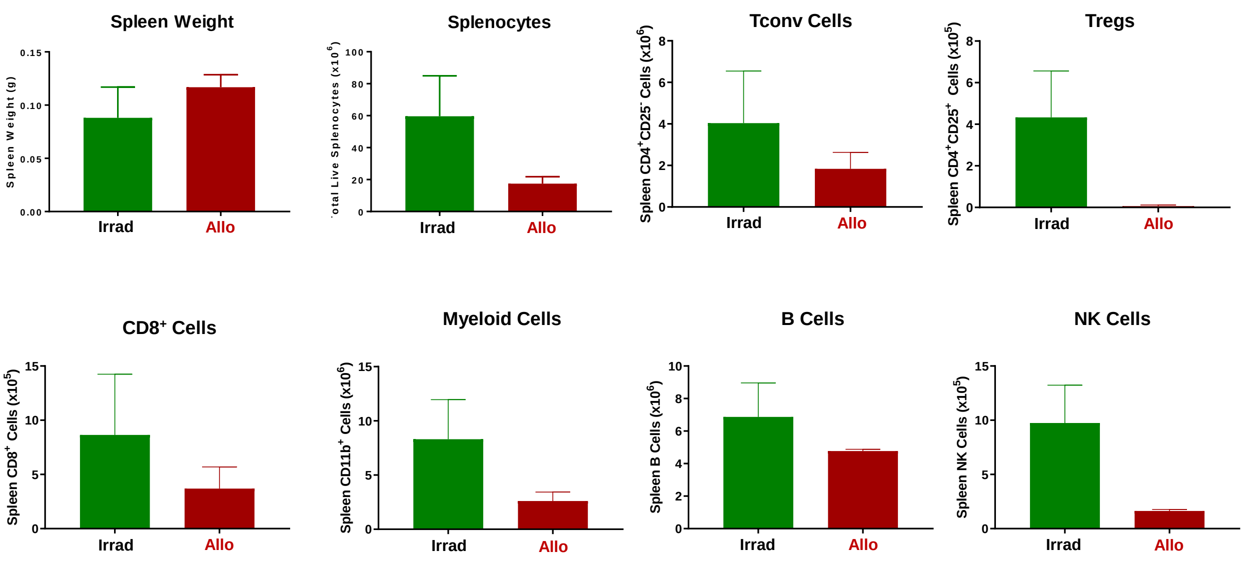

Figure S10. Spleen weight and immune cell analysis in CD1 mice engrafted with BM12 T cells housed at standard temperature. (A) Spleen weights, splenocyte numbers and spleen-residing immune cells were quantified at 4 weeks post T cell transfer for each mouse in the 2 groups described in Figure S6. Conventional T cells (Tconv cells; $CD4^+CD25^-$), regulatory T cells (Tregs; $CD4^+CD25^+$), CD8 T cells ($CD8^+$), Myeloid cells ($CD11b^+$), B cells ($CD19^+$) and NK cells ($CD335^+$) cells were quantified by flow cytometry as described in the Methods section 2.4. **(B)** Representative flow cytometry plot of syngeneic mice and **(C)** representative flow cytometry plot of allogeneic mice. The number of mice in the four groups are described in Figure S8. Data represent the mean ± SEM.
